# Non-Invasive Prediction Scores for Hepatitis B Virus- and Hepatitis D Virus-Infected Patients—A Cohort from the North-Eastern Part of Romania

**DOI:** 10.3390/microorganisms11122895

**Published:** 2023-11-30

**Authors:** Laura Iulia Grecu, Camelia Sultana, Mariana Pavel-Tanasa, Simona Maria Ruta, Mihaela Chivu-Economescu, Lilia Matei, Ramona Gabriela Ursu, Elena Iftimi, Luminita Smaranda Iancu

**Affiliations:** 1 Department of Preventive Medicine and Interdisciplinarity, Microbiology Discipline, Faculty of Medicine, “Grigore T. Popa” University of Medicine and Pharmacy, 700115 Iasi, Romania; lauraiuliagrecu@gmail.com (L.I.G.); ramona.ursu@umfiasi.ro (R.G.U.); luminita.iancu@umfiasi.ro (L.S.I.); 2 Department of Emerging Viral Diseases, “Stefan S. Nicolau” Institute of Virology, 030304 Bucharest, Romania; simona.ruta@umfcd.ro; 3 Virology Discipline, Faculty of Medicine, “Carol Davila” University of Medicine and Pharmacy, 020021 Bucharest, Romania; 4 Department of Immunology, Faculty of Medicine, “Grigore T. Popa” University of Medicine and Pharmacy, 700115 Iasi, Romania; iftimi.elena@yahoo.com; 5 Department of Cellular and Molecular Pathology, “Stefan S. Nicolau” Institute of Virology, 030304 Bucharest, Romania; mihaela.chivu@gmail.com (M.C.-E.);

**Keywords:** hepatitis D virus, hepatitis B virus, non-invasive scores, cirrhosis, hepatocellular carcinoma

## Abstract

Approximately 62–72 million people are infected worldwide with HDV. Patients with chronic hepatitis D (CHD) have a higher risk of developing cirrhosis or hepatocellular carcinoma (HCC) and an increased mortality rate compared to those with chronic hepatitis B (CHB). The stage of liver fibrosis or the risk of developing HCC can also be estimated by non-invasive scores, which are cost effective, easier to apply, and reproducible. In this study, we aimed to evaluate the predictive value of four non-invasive scores (FIB-4, APRI, AST/ALT ratio, and aMAP) in assessing severe fibrosis/cirrhosis and the presence of HCC in patients with HBV/HDV superinfection, as compared with HBV mono-infection. Our 8-year retrospective analysis revealed that HDV-infected patients had a 2–3 times higher risk of developing cirrhosis and HCC than HBV-mono-infected subjects. High AST and ALT baseline levels qualified as independent predictors for cirrhosis development in both groups. The following fibrosis scores, FIB-4, APRI score, and AAR, were significantly increased when cirrhosis was present at baseline and showed a good prediction for developing cirrhosis in the CHD group. The aMAP score, a risk predictor for HCC, showed significantly higher values in patients with HCC in both groups. Nonetheless, non-invasive scores should always be considered for monitoring patients with CHB and CHD, but only when associated with other diagnosis methods.

## 1. Introduction

Discovered more than 40 years ago by Mario Rizzetto, the hepatitis D virus (HDV) remains a challenge for both clinicians and researchers [1]. State-of-the-art studies reported that approximately 62–72 million people are infected worldwide with HDV [2]. HDV is a defective RNA virus that depends on the presence of hepatitis B virus (HBV) to infect hepatocytes and to assemble into new virions after replication.

Hepatitis B remains a major health problem and economic burden worldwide, as the World Health Organization (WHO) estimated that 296 million people had chronic hepatitis B infection (CHB), which, causing liver cirrhosis and hepatocellular carcinoma, was associated with an increased mortality that resulted in 820,000 deaths globally in 2019 [3]. Thus, the WHO set as a global target for 2030 a 65% reduction in viral hepatitis-related deaths together with a 90% reduction in the incidence of viral hepatitis infections compared to the reported rates for 2015 [4]. In the European Union (EU)/European Economic Area (EEA) and the United Kingdom (UK), 4.7 million individuals were estimated to have CHB, which resulted in 43,000 deaths in 2019 [3]. Consequently, the European Region is considered low-prevalence for HBV (overall score below 2%), however, with marked geographical differences: higher estimated prevalence in eastern and southern Europe and lower in the western, central, and northern countries [5,6]. A recent systematic study predicted that the highest HBV prevalence scores in the general European population were registered in Cyprus with 6.68% (5.76–7.60%), followed by Romania with an estimate of 2.87% (2.74–3.01%) [7]. Importantly, a high endemicity for HBV infection was reported in Romania before the introduction of universal childhood vaccination in 1995, when the incidence was 43 per 100,000 people and decreased to 8.5 per 100,000 in 2004 [8], and reached the value of 0.09 per 100,000 in 2021, when the target of 90% hepatitis B vaccination coverage at birth was also reached [9].

The HBV/HDV co-infection (simultaneous infection with both viruses) causes a more rapid progression to severe liver damage and fulminant hepatitis as compared to HBV mono-infection, while HBV/HDV superinfection (HDV infection of an HBV carrier) leads to chronicity in over 90% of cases [10,11]. Patients with chronic hepatitis D (CHD) have a 2-fold higher risk of developing cirrhosis, a 3-fold higher risk of developing HCC, and a 2-fold higher mortality rate compared to chronic hepatitis B (CHB) [12]. A recent large retrospective study, including three European countries (Croatia, Romania, and Spain), reported an overall HBV/HDV co-infection rate of 9% in Romania for samples collected between 1 January 2019 and 30 June 2019, with 3.5% of the tested HBV-infected patients originating from the northern–eastern region [13]. Importantly, the report also indicated that the treatment status was reported only for 89% of the HBV patients, with 51% of patients being treated and 89% achieving viral suppression.

The progression of liver disease is also influenced by the HBV genotypes. For Romania, little data are available, as one study from 2008 reported the frequencies of HBV genotypes being A, D, or mixed (6%, 67%, and 27%, respectively) [14]. Importantly, a more recent study from 2019, where HBV-infected patients from four counties in Romania (Iasi, Calarasi, Sibiu, and Suceava) were tested and supplementary reported the HBV genotype F [15]. In this context, the stage of liver fibrosis can be determined via biopsy, imaging-based techniques (ARFI—Acoustic Radiation Force Impulse Imaging or SWE—Shear Wave Elastography), transient elastography (FibroScan) [16], or by non-invasive scores which are cost effective, easier to apply, and reproducible. Advanced liver fibrosis can be assessed via APRI (aspartate transaminase (AST) to platelet ratio index), AST/alanine transaminase (ALT) ratio, or FIB-4 (fibrosis-4 index), and the risk of developing HCC by using aMAP (age-male-ALBI (albumin–bilirubin)-platelets) score. These non-invasive scores have acceptable specificity and sensitivity compared to the “gold standard” represented by liver biopsy in HCV cohorts [17,18] but with lower performance in hepatitis B or D [19,20].

FIB-4 was described as a score with modest performance in hepatitis B or D fibrosis assessment (AUROC [0.7; 0.8]) [19,20]. For the APRI score, succeeding studies have shown moderate accuracy in HBV-related cirrhosis (AUROC [0.66; 0.72]) [21,22]. As for HDV, APRI proved to be a poor predictor (AUROC 0.65), but this could be improved by adjusting the cut-off values [23]. AST/ALT ratio (AAR) was correlated with cirrhosis in patients with hepatitis B [24]. aMAP score was further described as an independent risk predictor for HCC occurrence and mortality in HBV-infected patients [25,26].

Recently updated EASL (European Association for the Study of the Liver) guidelines recommend that new non-invasive tests (NITs) should permit diagnostic prediction and, beyond this, allow for the monitoring of liver fibrosis progress and its complications [27]. In this study, we aimed to evaluate the predictive value of four non-invasive scores in assessing severe fibrosis/cirrhosis and the presence of HCC in patients with HBV/HDV superinfection, as compared with HBV mono-infection.

## 2. Materials and Methods

### 2.1. Patients and Biological Samples

We retrospectively examined the medical records from all the patients diagnosed with chronic hepatitis B and chronic hepatitis D admitted to university hospitals in Iasi, Romania (“St. Spiridon” Emergency Clinical County Hospital and “St. Parascheva” Clinical Infectious Diseases Hospital from Iasi), from 2012 to 2019. These two hospitals are the most representative from the north-eastern (NE) part of Romania and have the highest addressability of patients with viral hepatitis from this region, as also reported by Nardone et al. [13]. Importantly, “St. Spiridon” Emergency Clinical Country Hospital is the university hospital that provides care, according to the internal statistical data, to 67% of patients from Iasi County and 33% of patients from the NE region, characterized by a population exceeding 3.7 million residents (representing more than 17% of the total population of Romania). This university hospital also comprises the Regional Institute of Gastroenterology and Hepatology, which is in close collaboration with the local liver transplant team. Importantly, the “St. Parascheva” Clinical Infectious Diseases Hospital is the largest hospital for infectious diseases in this region. The study was approved by the institutional ethics committees of the University and hospitals as well. We collected demographical, paraclinical, and clinical data from 2319 HBV-infected patients.

The diagnosis of fibrosis and cirrhosis was based on three categories of parameters: biochemical markers (elevated levels of ALT, AST, gamma-glutamyl transferase, total bilirubin, alpha-fetoprotein, prothrombin time (international normalized ratio (INR)) and decreased platelet (PLT) count, and serum albumin level), virological determinations using immunological markers (the presence of HBsAg, HBeAg, total anti-HBc, anti-HBe, and anti-HDV antibodies), nucleic acid tests (polymerase chain reaction (PCR) for HBV-DNA and RT-PCR for HDV-RNA), and imagistic criteria (ultrasound evaluation of the parenchymal texture, surface nodularity, volumetric changes, and liver edge). The immunological markers were routinely investigated using commercial in vitro diagnostic kits suitable for the Architect 2000 and the Cobas automated platforms. For objective reasons related to hospital logistics, the assessment of viral loads for HBV (HBV-DNA—Bosphore^®^ HBV Quantification-Detection in vitro diagnostics kit (Istanbul, Turkey): analytical detection limit 1 IU/mL, where 1 IU = 4.5 ± 0.2 copies; linear range 10^1^–10^9^ IU/mL; genotype detection: HBV genotypes A-H; no cross-reactivity with highly positive Cytomegalovirus, Epstein–Barr Virus, MTBC, Parvovirus B19, BK Virus samples; coefficient of variations for intra-assay and inter-assay variabilities: 0.23% and 0.93%, respectively) on Roche LightCycler; and HDV (HDV-RNA—LightMix Kit HDV (Berlin, Germany) on Roche LightCycler (analytical detection limit 10 copies/mL; linear range 10^1^–10^6^ copies/mL; genotype detection: HDV genotype 1) was performed via an outsourced laboratory.

HCC was diagnosed according to the EASL—Clinical Practice Guidelines: Management of hepatocellular carcinoma (mass/nodule at imaging, Computed tomography scan, or Magnetic resonance imaging followed by biopsy) [28]. 

Patients’ inclusion criteria were (i) age > 18 years; (ii) confirmed HBV infection by PCR; and (iii) at least two hospitalization records at minimum 6-month intervals. Patients’ exclusion criteria were (i) incomplete paraclinical data set; (ii) HCV or HIV co-infection; (iii) autoimmune diseases; and (iv) chronic alcohol consumption. Based on the above criteria, we selected for our study 436 patients for the chronic HBV infection (CHB) cohort and 166 cases for the chronic HBV/HDV superinfection (CHD) cohort. 

Patient diagnosis, monitoring, and selection for treatment were performed following the EASL Clinical Practice Guidelines on the management of chronic HBV [29,30] or HDV infection [31]. The age of patients at diagnosis was 56 ± 11 years for the CHB cohort and 52 ± 12 years for the CHD cases. In the present study, 110 (25.2%) patients in the CHB group and 60 (36.1%) patients in the CHD group met the EASL recommendations for receiving antiviral treatment during follow-up. For instance, the EASL guidelines recommend that chronically HBV-infected patients be considered for treatment when the following criteria are met: (i) HBV DNA levels above 2000 IU/mL, (ii) serum ALT levels above the upper limit of normal (ULN), and (iii) severity of liver disease assessed by liver biopsy (or non-invasive markers) showing moderate to severe active necroinflammation [29,30]. In our cohorts, the majority of patients eligible for treatment received monotherapy with nucleoside analogs or Interferon as follows: for the CHB group, 40 (36.4%) patients received Interferon monotherapy, 64 (58.2%) received Entecavir, 2 (1.8%) received Tenofovir, and only 4 (3.6%) received at least two antivirals (Entecavir combined with Interferon, Adenofovir, or Tenofovir); for the CHD group, 42 (70%) patients received Interferon (Roferon), 13 (21.7%) received Entecavir, 1 (1.7%) received Tenofovir in monotherapy, and only 4 subjects (6.7%) received at least two antivirals (Entecavir combined with Interferon or Adenofovir). For the majority of patients, the therapy was initiated within the first year of diagnosis (median time 1 year; time range: 0–5 years for CHB and 0–6 years for CHD).

### 2.2. Non-Invasive Biological Scores

Four non-invasive scores (FIB-4, APRI, AST/ALT ratio (AAR), and aMAP) were calculated for each patient at the first registered hospitalization according to the recommended formulas (Table 1). Since we included in the study only the patients with at least two hospitalizations, we were able to perform a follow-up study and assess the efficacy of non-invasive scores in predicting the outcome of CHB or CHD patients.

### 2.3. Statistical Analysis

IBM SPSS version 20 was used for data collection and analysis, and statistically significant results were considered at a confidence level of at least 95%. The Shapiro–Wilk test and histogram were used to examine the normal distribution of data. For descriptive statistics, data with a normal distribution were represented as mean and standard deviation, and non-parametric data as median and interquartile range (IQR). For comparative statistics, χ^2^ test, independent T-test, and non-parametric statistical tests, such as the Mann–Whitney U or Kruskal–Wallis tests were used. Binary logistic regressions were used to assess the associations between different demographic, paraclinical parameters or NITs and the outcome. Univariate and multivariate proportional hazards Cox regressions were used to identify independent predictors for cirrhosis or HCC and to compare the risk of cirrhosis and HCC in both groups. More precisely, after identifying the baseline independent predictors for cirrhosis or HCC using the Univariate Cox regression analysis, we selected the most important confounders (including comorbidities) reported by other studies and known to influence the course of the liver disease for adjustment using a Multivariate Cox regression analysis. The area under the receiver operating characteristics (AUROC) was used for evaluating the diagnostic accuracy of different non-invasive scores for cirrhosis and hepatocellular carcinoma. In order to adjust the NITs’ prediction values for possible confounders, we also included in our analysis the baseline independent predictors for cirrhosis and HCC and the comorbidities known to influence the evolution of the liver disease (diabetes, essential hypertension, obesity, smoking status, ischemic cardiac disease, or non-alcoholic fatty liver disease (NAFLD)) [36,37,38]. Specifically, for controlling the potential confounding variables, we first performed a binominal regression of independent predictors and comorbidities, and the obtained adjusted probabilities were further used to adjust the AUROC curves. Kaplan–Meier curves and Log-rank test were used to calculate the cumulative survival for cirrhosis and HCC in both groups (CHB and CHD).

## 3. Results

### 3.1. General Characteristics of the Patients

**Figure 1:**
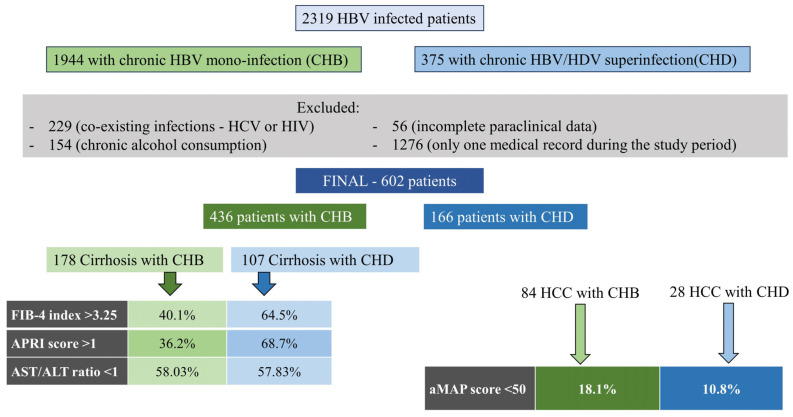
General design of the study.

A total of 2319 patients, of whom 1944 had CHB and 375 had chronic HBV/HDV superinfection (CHD), were identified in the investigated period. An amount of 229 cases was excluded because of other co-existing viral infections (HCV or HIV); 154 patients were excluded because of chronic alcohol consumption, and 56 subjects because of incomplete paraclinical data. Also, 1276 patients were excluded as they had only one medical record during the study period. In the end, the study population included 602 patients, of whom 436 had CHB and 166 had CHD (Figure 1).

The median age for the entire cohort of patients was 57 years, with a predominance of male patients (*n* = 372, 61.8%) and urban origin (*n* = 371, 61.6%). The prevalence of HDV among HBV-infected patients was 16.2%. The CHB group had older patients compared to the CHD group (*p* < 0.001), maintaining the predominance of male gender (64.9% of CHB vs. 53.6% of CHD) and urban origin (60.3% of CHB vs. 65.1% of CHD) in each group. The analysis of various biochemical markers identified that the CHB group had higher cholesterol levels than the CHD group (*p* = 0.016). On the other hand, the CHD group presented increased alpha-fetoprotein (AFP) (*p* < 0.001), AST (*p* < 0.001), ALT (*p* < 0.001), and GGT levels (*p* = 0.048) and lower PLT blood count (*p* < 0.001) when compared with the CHB group (Table 2).

The analysis of comorbidities at baseline further revealed a higher frequency of diabetes (17.4% vs. 10.2%, *p* = 0.032) and essential hypertension (30.7% vs. 19.3%, *p* = 0.006) in the CHB group compared to CHD (Table 3). For the other identified comorbidities, including obesity, smoking status, ischemic cardiac disease, or non-alcoholic fatty liver disease (NAFLD), no significant differences were observed between the two distinct groups of patients (CHB and CHD).

At the first registration in the study, 40.8% of HBV-mono-infected patients already had a diagnosis of cirrhosis and 19.3% had a diagnosis of HCC; meanwhile, among HDV patients, 64.5% already had cirrhosis and 16.9% had HCC. According to the baseline characteristics, the patients were stratified into three categories: (i) patients without any sign of end-stage liver disease (neither cirrhosis nor HCC), (ii) a cohort with current cirrhosis but without HCC, and (iii) patients with HCC (Table 4).

The analysis of baseline comorbidities in relation to the above-mentioned categories (Table 5) revealed that the NAFLD (*p* < 0.001), obesity (*p* = 0.034), and hypertension (*p* = 0.035) diagnoses were less frequent in the cirrhosis and HCC subgroups compared to the no cirrhosis nor HCC subgroup within the CHB cohort. A similar trend was observed for the NAFLD diagnosis also in the CHD cohort. Importantly, all the diabetes cases had either cirrhosis or HCC within the CHD cohort (*p* = 0.024).

### 3.2. Independent Predictors for Cirrhosis Development in CHB vs. CHD Patients

At baseline, 178 (40.8%) of CHB and 107 (64.5%) of CHD patients already had a diagnosis of cirrhosis. CHD patients with cirrhosis had higher levels of AFP (*p* = 0.012), AST (*p* = 0.001), ALT (*p* < 0.001), and lower levels of PLT (*p* < 0.001) when compared to CHB patients with cirrhosis. Also, the CHB group with cirrhosis had a higher median age than the CHD group with cirrhosis (*p* = 0.003) (Supplementary Table S1).

A binominal logistic regression was performed to ascertain the association of baseline paraclinical data (gender, age, rural/urban origin, cholesterol level, AFP, AST, ALT, GGT, BLBt, Albumin, PT (INR), and PLT levels) with the likelihood to have cirrhosis. In the CHB group, only low levels of albumin (0.719, 95% CI [0.545, 0.948], *p* = 0.019) and low levels of platelets (0.988, 95% CI [0.985, 0.992], *p* < 0.001) were significantly associated with the presence of cirrhosis. In the CHD group, none of the paraclinical data was associated with the presence of cirrhosis at baseline. 

During a mean follow-up of 4.1 years, 33 HBV-mono-infected patients developed cirrhosis, with an incidence of cirrhosis of 3.96 per 100 patient years. On the other hand, 12 HDV-infected patients developed cirrhosis during a mean follow-up of 4.7 years, with an incidence of cirrhosis of 8.73 per 100 patient years, resulting in HDV-infected patients having a 2.33 times higher risk than HBV to develop cirrhosis (Cox regression univariate analysis, 95% CI [1.20–4.51], *p* = 0.012).

A binominal logistic regression was performed In order to identify the association between paraclinical data and the probability of developing cirrhosis. Only high values of AST (1.020, 95% CI [1.003, 1.039], *p* = 0.024) at baseline were associated with the likelihood of developing cirrhosis in the CHB group during follow-up.

Univariate proportional hazards Cox regression was performed in order to detect independent predictors for developing cirrhosis. High AST and ALT levels in both groups, high GGT levels only in the CHD group, high AFP levels in the CHB group, and low PLT levels in both groups qualified as independent predictors for developing cirrhosis during follow-up (Supplementary Table S2). After adjusting the baseline characteristics for possible confounders (age, gender, diabetes, NAFLD, obesity, smoking status, essential hypertension, or history of ischemic cardiac disease) in a multivariate Cox regression analysis, the same independent predictors were found. 

### 3.3. The Significance of the Non-Invasive Scores in Cirrhosis Prediction

FIB-4 and APRI scores calculated at first admission predicted higher rates of advanced fibrosis in the CHD group than in the CHB group (Table 6):

All three scores were significantly increased (FIB-4 *p* < 0.001, Z = −10.84; APRI score *p* < 0.001, Z = −10.11; and AAR *p* < 0.001, Z = −6.4) when cirrhosis was present at baseline. FIB-4 and APRI scores showed significantly higher rates of cirrhosis in CHD patients (*p* = 0.005, Z = −2.78 and *p* < 0.0001, Z = −5.12). Using binary logistic regression, the high FIB-4 score (1.233, 95% CI [1.098, 1.385], *p* < 0.001) and AAR (1.441, 95% CI [1.020, 2.035], *p* = 0.038) were associated with the presence of cirrhosis at baseline only in the CHB group. 

During follow-up, high levels of FIB-4 at baseline were associated with a higher risk of developing cirrhosis (1.725, 95% CI [1.122, 2.652], *p* = 0.013) in the CHB group, while high levels of APRI score at baseline with a higher risk to develop cirrhosis (13.764, 95% CI [1.146, 165.248], *p* = 0.039) in the CHD group.

**Figure 2:**
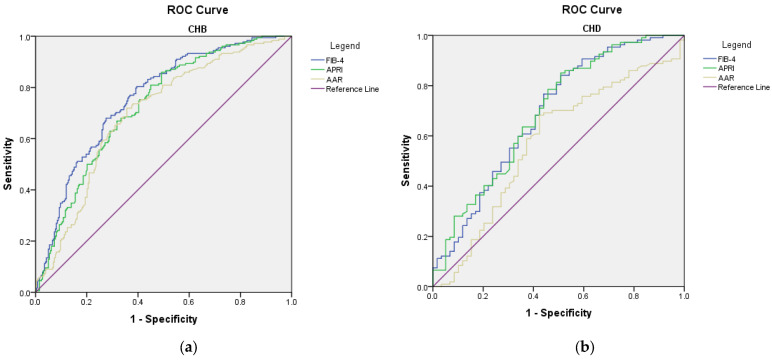
ROC curves of non-invasive scores for predicting the diagnosis of cirrhosis: (**a**) CHB; (**b**) CHD.

**Figure 3:**
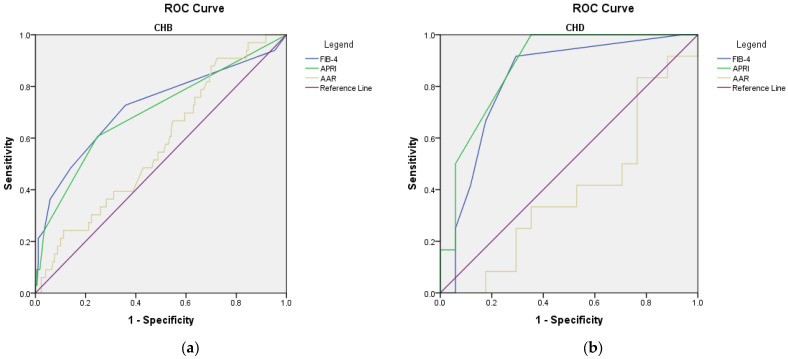
ROC curves of non-invasive scores in assessing the risk of developing cirrhosis during follow-up: (**a**) CHB; (**b**) CHD.

The receiver operating characteristic analysis showed that all three scores for fibrosis failed to predict the present diagnosis of cirrhosis (AUROC < 0.800) (Figure 2). Still, in the CHD group, FIB-4 and APRI scores had good performance in predicting the risk of cirrhosis during the follow-up (AUROC 0.824 (*p* = 0.003) and 0.877 (*p* = 0.001), respectively) (Figure 3). Sensitivity and specificity for FIB-4 as a predictor for cirrhosis in CHD are 91.7% and 70.6, respectively, and PPV 58.1%. Sensitivity and specificity for APRI as a predictor for cirrhosis in CHD is 100% and 64.7% and PPV 62.3%.

**Figure 4:**
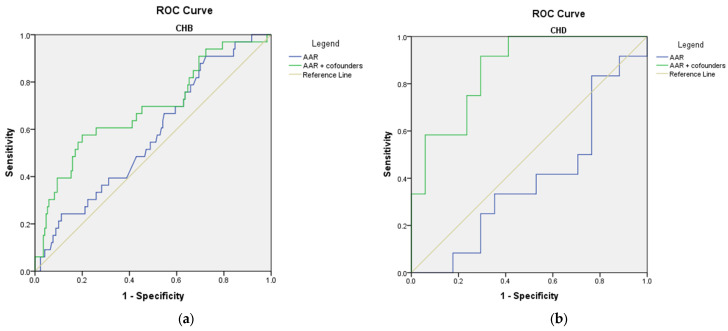
ROC curves of the non-invasive AAR score adjusted for confounders in assessing the risk of developing cirrhosis during follow-up: (**a**) CHB; confounders: age, gender, levels of AFP, diabetes, NAFLD, obesity, smoking status, hypertension I, history of ischemic cardiac disease; (**b**) CHD; confounders: age, gender, levels of GGT, diabetes, NAFLD, obesity, smoking status, hypertension I, history of ischemic cardiac disease.

When adjusting for various possible confounders (age, gender, levels of AFP, ALT, or GGT, diabetes, NAFLD, obesity, smoking status, essential hypertension, or history of ischemic cardiac disease), no significant changes in the AUROC values were achieved for the FIB-4 and APRI scores in predicting the risk of developing cirrhosis (Supplementary Figures S1 and S2, and Supplementary Table S3). Interestingly, the predictive role of AAR was majorly improved after adjusting for the above-mentioned confounders, especially in the CHD group (AUROC 0.863, *p* = 0.001; Figure 4 and Supplementary Table S3).

### 3.4. Independent Predictors for the HCC Development in CHB and CHD Patients

A total of 84 (19.3%) patients with CHB and 28 (16.9%) patients with CHD had already been diagnosed with HCC at the first registration in this study. According to their medical records, 31 CHB patients and 5 CHD patients with HCC were not previously diagnosed with cirrhosis. Among HCC patients, the CHD subjects were younger (*p* = 0.022) and had lower PLT levels when compared to the CHB cases (*p* = 0.001) (Supplementary Table S4).

A binominal logistic regression was performed to determine the association of paraclinical data (gender, age, rural/urban origin, cholesterol level, AFP, AST, ALT, GGT, BLBt, Albumin, PT (INR), and PLT levels) at baseline with the likelihood to have HCC. Male gender (1.917, 95% CI [1.007, 3.646], *p* = 0.047), older age (1.034, 95% CI [1.005, 1.063], *p* = 0.019), higher AFP levels (1.004, 95% CI [1.000, 1.007], *p* = 0.024), and higher AST levels (1.009, 95% CI [1.003, 1.015], *p* = 0.006) were associated with a higher probability to have a diagnosis of HCC at baseline in the CHB group. In the CHD group, higher AFP levels (1.011, 95% CI [1.003, 1.019], *p* = 0.006) were associated with a higher probability of having a diagnosis of HCC at baseline.

Regarding the HBV-mono-infected patients without HCC at baseline, 30 new cases of HCC were recorded during a mean follow-up of 4.7 years, meaning an incidence of HCC of 1.87 per 100 patient years. Of the HDV patients without HCC at baseline, 32 new cases of HCC were recorded during a mean follow-up of 4.5 years, meaning an incidence of HCC of 5.69 per 100 patient years. Cox regression univariate analysis indicates that HDV-infected patients have a 3.03 (95% CI [1.84–4.99], *p* < 0.001) higher risk of developing HCC than HBV-mono-infected patients.

A binominal logistic regression was performed to identify the association between paraclinical data and the probability of developing HCC. Higher AFP levels (1.015, 95% CI [1.005, 1.025], *p* = 0.003) and lower PLT levels (0.988, 95% CI [0.980, 0.995], *p* = 0.001) at baseline were associated with a higher probability to develop HCC in CHB group during the follow-up. In the CHD group, being older (1.081, 95% CI [1.019, 1.417], *p* = 0.010) and lower albumin levels (0.544, 95% CI [0.323, 0.917], *p* = 0.022) were associated with a higher probability to develop HCC during the follow-up.

Univariate Cox regression was performed to identify independent predictors for HCC at baseline, testing the same parameters as for the patients with cirrhosis. In the CHB group, high AFP and AST levels and low PLT levels were found to be predictors for HCC during follow-up. In the CHD group, older age, high AFP levels, and low albumin qualified as independent predictors for HCC (Supplementary Table S5). After adjusting the baseline characteristics for possible confounders, like age, gender, the presence of diabetes, NAFLD, obesity, smoking status, essential hypertension, or history of ischemic cardiac disease at baseline, only high AFP and PLT levels in the CHB group, and age and low albumin in the CHD group remained independent predictors for HCC.

### 3.5. Non-Invasive Scores Prediction for HCC

The aMAP score calculated at first admission predicted lower rates of low risk of HCC in the CHD group than in the CHB group (Table 7):

aMAP score showed significantly higher values in patients with HCC in both groups (*p* = 0.004 for the CHB group; *p* = 0.040 for the CHD group). Upon binary logistic regression, aMAP score (1.038, 95% CI [1.010, 1.067], *p* = 0.008) was associated only in the CHB group with the presence of HCC at the baseline. On the other hand, a high aMAP score for the CHB (1.072, 95% CI [1.024, 1.123], *p* = 0.003) and CHD (1.092, 95% CI [1.029, 1.158], *p* = 0.004) groups were associated with a higher likelihood to develop HCC during the follow-up period.

**Figure 5:**
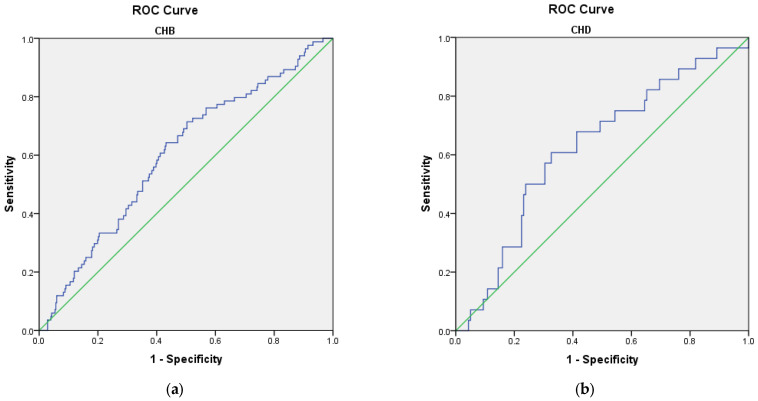
ROC curves of non-invasive scores for predicting the diagnosis of HCC: (**a**) CHB; (**b**) CHD. Note: aMAP line—blue; reference line—green.

**Figure 6:**
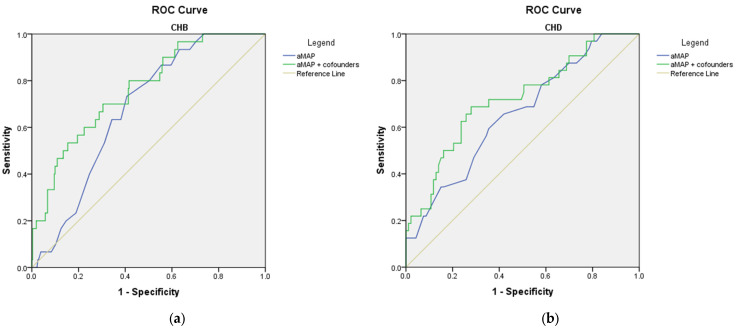
ROC curves of the non-invasive aMAP score adjusted for confounders in assessing the risk of developing HCC during follow-up: (**a**) CHB; confounders: levels of AST and AFP, diabetes, NAFLD, obesity, smoking status, essential hypertension, history of ischemic cardiac disease; (**b**) CHD; confounders: levels of AST AFP, diabetes, NAFLD, obesity, smoking status, essential hypertension, history of ischemic cardiac disease.

aMAP score failed to predict HCC diagnosis or the risk of developing HCC during follow-up for both CHD and CHB groups (AUROC < 0.700) (Figure 5 and Figure 6), even when adjusting for possible confounders (CHB: levels of AST and AFP, diabetes, NAFLD, obesity, smoking status, essential hypertension, history of ischemic cardiac disease; CHD: levels of AST AFP, diabetes, NAFLD, obesity, smoking status, essential hypertension, or history of ischemic cardiac disease; Figure 6 and Supplementary Table S6).

### 3.6. Survival Curves for CHB and CHD Patients

**Figure 7:**
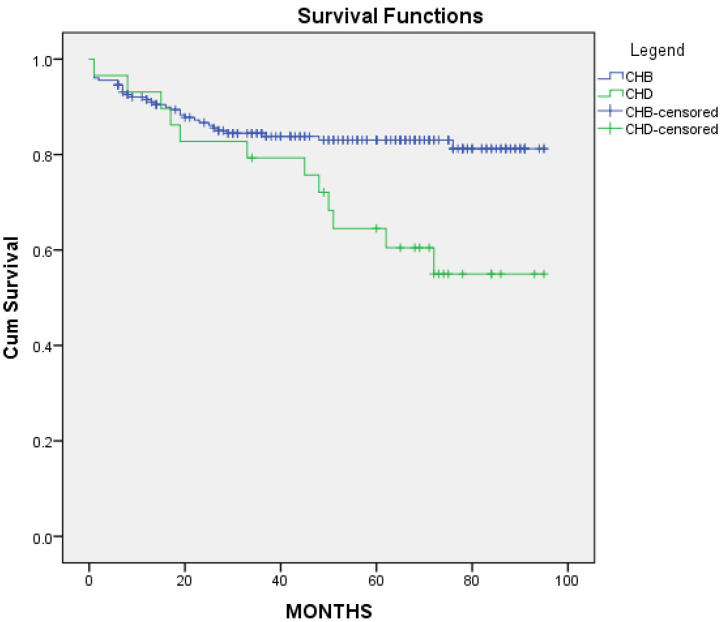
Cirrhosis-free survival curves for CHB and CHD cohorts.

The cumulative event-free survival probability for cirrhosis was lower in the CHD group, with a median of 69 months IQR [57,81] vs. 80 months IQR [76,85] in the CHB group (*p* < 0.001) (Figure 7).

**Figure 8:**
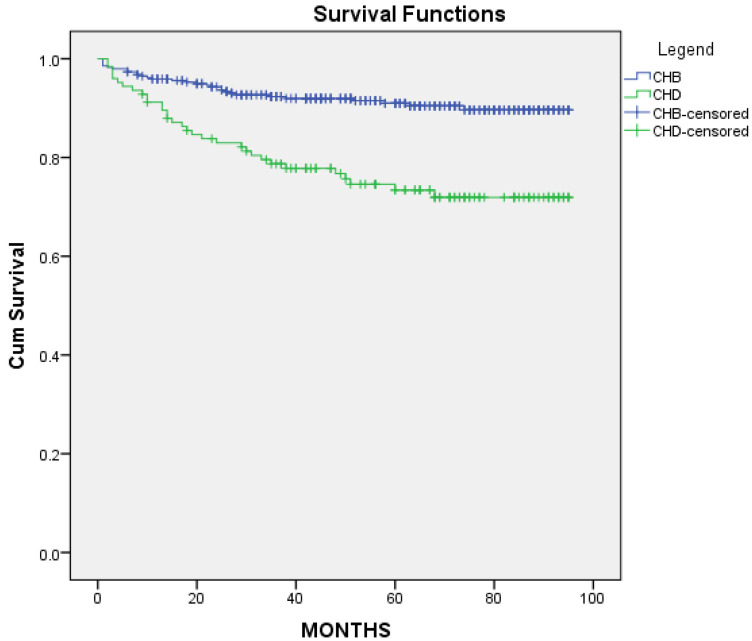
HCC-free survival curves for CHB and CHD cohorts.

The mean survival time HCC free for the CHB group was 88 months IQR [85,90] and 74 months IQR [69,81] (*p* < 0.001) for the CHD group (Figure 8).

### 3.7. Assessment of Virological Status on Cirrhosis and HCC

For the majority of patients, virological status was assessed based on virological markers such as HBsAg levels and the presence or absence of HBeAg. Several studies have proved that HBsAg may be a reliable tool to predict the outcome of liver disease depending on the HBeAg status [39,40] or to assess the response to treatment [41,42,43,44]. In predicting the outcome of the disease, the decrease in HBsAg level with at least 1 log IU/mL shows the improvement in host immune control, and only in HBeAg-negative patients was a level of HBsAg higher than 1000 IU/mL associated with a higher risk of developing cirrhosis, which proved to be a better predictor for HCC than HBV-DNA level [45]. In other studies, lower levels of HBsAg were associated with advanced fibrosis and cirrhosis in HBeAg-positive patients [40,46]. HBeAg is often used to assess the stage of the liver disease or to establish the endpoint treatment [43] but was also seen to be correlated with the virological response in patients with NUC (nucleos(t)ide analogs) therapy [41].

The monitoring of HBsAg dynamics during antiviral treatment for HBV infection proved to be reliable, especially when there is a rapid drop in HBsAg level [43,45] or lower levels at the beginning and during treatment with NUC therapy [47]; or when very low levels such as ≤10 IU/mL can predict higher rates of post-treatment HBsAg loss after IFN therapy [47]; or, when there is no potential to test the viral load, HBsAg quantification can reflect HBV-DNA levels or predict the chances to obtain undetectable HBV-DNA during treatment and follow-up [44,48].

In our study, the HBsAg levels were recorded, and no significant differences were observed at baseline between the CHB (mean 3605.1 STD 2647.9) and CHD group (mean 4040.5 STD 2712.9) (*p* > 0.05—independent *t*-test). There were no significant differences (CHB *p* = 0.788 and CHD *p* = 0.224 ‡ Kruskal–Wallis Test) at the baseline for the three categories: (i) patients without any sign of end-stage liver disease (neither cirrhosis, nor HCC), (ii) a cohort with current cirrhosis but without HCC, and (iii) patients with HCC (for CHB group: i—median 3562 IQR [867–5440], ii—median 3578 IQR [501.5–5868.3], and iii—median 3489 IQR [2037–6452.3]; for CHD group: i—median 3252 IQR [2336–8646.8], ii—median 3872 IQR [1581–6055], and iii—median 1560 IQR [1444–1570]). There was no association between the HBsAg levels and the presence of cirrhosis or HCC at the baseline, nor the development of these two conditions during follow-up.

When assessing the role of the virological status (including both HBsAg levels and HBeAg status) in adjusting the predictive value of the non-invasive scores for developing cirrhosis or HCC, almost no significant contributions were noticed. For instance, none of the investigated scores or in association with virological status and/or confounders were able to accurately predict the risk of developing cirrhosis in CHB patients (Supplementary Figure S3). However, including the virological status significantly increased the predictive value for cirrhosis of AAR and confounders in the CHD group (AUROC increased from 0.863 to 0.951, *p* < 0.001; Supplementary Figure S4). Importantly, the virological status had no influence on the predictive role of aMAP for HCC in both cohorts (Supplementary Figure S5).

### 3.8. Assessment of Antiviral Treatment on Cirrhosis and HCC

In the present study, only 110 (25.2%) patients in the CHB group and 60 (36.1%) patients in the CHD group received antiviral treatment during follow-up. Almost all the patients in the treatment group received monotherapy with nucleoside analogs or Interferon as detailed in the Materials and Methods section.

For assessing the effect of therapy in adjusting the predictive value of the investigated non-invasive scores for developing cirrhosis or HCC, extensive ROC analyses were performed and the AUROC values were computed as detailed in Table 8 and Table 9.

**Figure 9:**
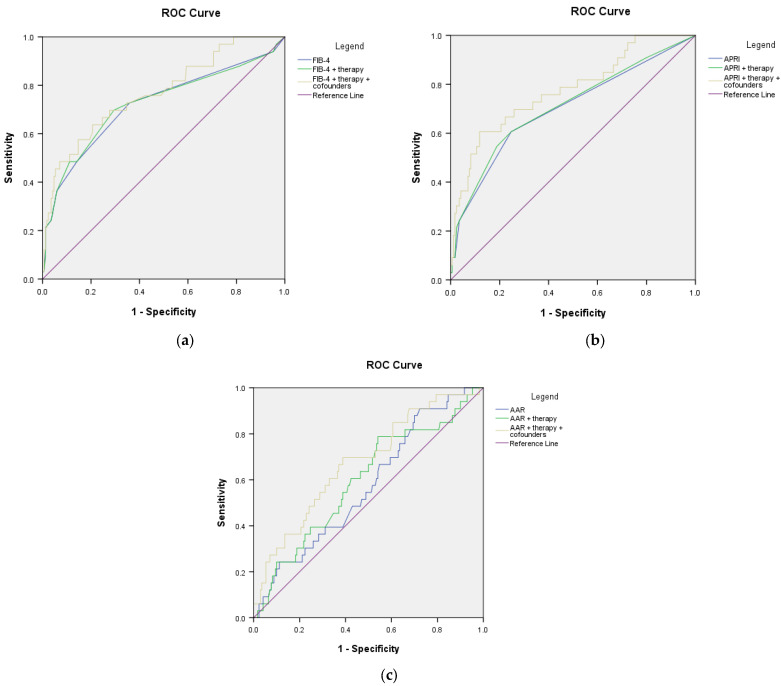
ROC curves of non-invasive scores adjusted for antiviral therapy for predicting the risk of developing cirrhosis in CHB: (**a**) FIB-4; (**b**) APRI; (**c**) AAR.

**Figure 10:**
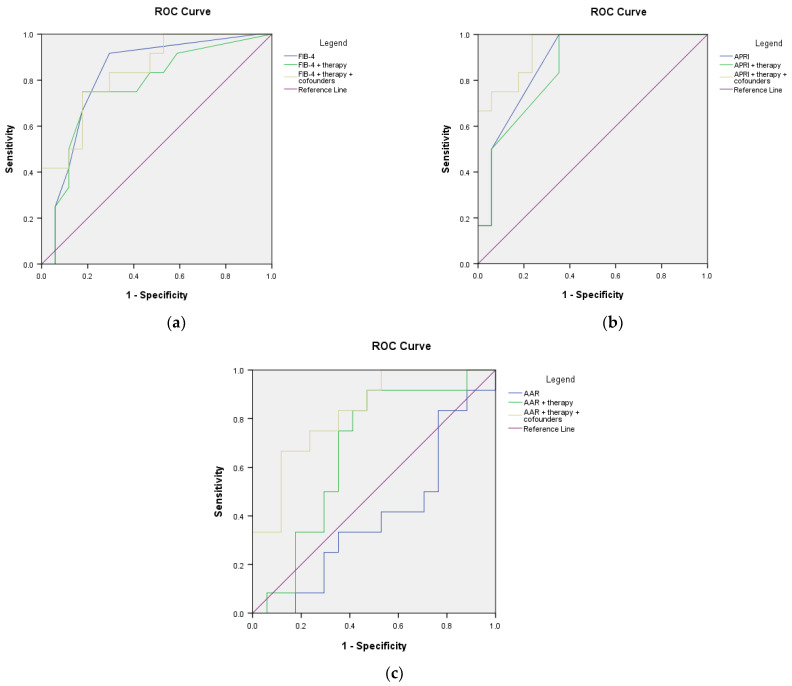
ROC curves of non-invasive scores adjusted for antiviral therapy for predicting the risk of developing cirrhosis in CHD: (**a**) FIB-4; (**b**) APRI; (**c**) AAR.

In the CHB cohort, none of the markers (FIB-4, APRI, or AAR) at baseline, either alone or in association with therapy and/or the previously investigated confounders, were able to accurately predict the risk of developing cirrhosis (AUROC values below 0.800, Figure 9 and Table 8). Similarly, including therapy in the analysis of the CHD group did not bring any significant improvement in predicting the risk of developing cirrhosis. Interestingly, therapy caused a marginally positive effect on the AAR score. However, it did not yield a significant AUROC value (Figure 10 and Table 8).

When assessing the role of therapy in adjusting the predictive value of aMAP for developing HCC, the ROC analysis failed to prove any positive effects (Table 9).

Additionally, in both CHB and CHD cohorts, there were no significant differences in the rate of developing cirrhosis or HCC between those who received antiviral therapy and those who did not (*p* > 0.05). Based on these observations, we could conclude that therapy had a marginal role in influencing the predictive values of the investigated non-invasive scores in our CHB and CHD cohorts.

## 4. Discussion

By the end of 2019, routine Hepatitis B vaccination for all infants or children aged 1–12 years (universal HepB) was administered in 50 countries in the EU/EEA, accounting for 94%. Three countries (Denmark, Finland, and Iceland) did not implement the universal HepB in their routine childhood immunization, while 23 countries (43%) implemented the universal HepB birth dose (HepB-BD) for all newborns [49]. Romania adhered to the universal HepB-BD policy in 1995, and since then, the incidence decreased from 43 to 0.09 per 100,000 people in 2021.

The prevalence of HDV among HBV infected patients in our study was 16.2%, which was a value found in the range reported by other Romanian cohorts of 9–23.1% [13,50,51,52], and comparable with various reports from Europe [53]. In our study, we have a predominance of males at 61.8%; gender is described in other studies as an independent factor associated with a more severe prognosis for hepatitis D [36]. Importantly, there is a rich literature that reported male gender as an independent predictor for liver disease progression and even emphasized a higher prevalence for an unfavorable course of liver disease in male cohorts [25,54,55,56]. Hepatitis D virus infection is known to cause the most severe form of viral hepatitis with the worst prognosis [57]. The pathophysiological impact of the association of HDV infection is reflected in the paraclinical data in our study: CHD patients had higher median levels of transaminases, GGT, and AFP and lower levels of PLT blood count compared with CHB cases.

To avoid invasive diagnostic procedures with a high risk of complications, non-invasive scores were developed in order to determine the liver damage and predict morbidity. FIB-4 and APRI designed for predicting cirrhosis (advanced liver fibrosis) had higher median levels in our study at the baseline in the CHD cohort. AST/ALT ratio levels were similar in the two cohorts.

The importance of using NITs has been suggested for many years now, even mentioned in the recently updated EASL guidelines, due to their cost efficiency, reproducibility, and applicability [27]. These tests are aimed at facilitating diagnostic prediction and offering a flexible tool for monitoring the progression of liver fibrosis and its complications [27]. There are several studies in the literature that have approached to investigate the effectiveness of these scores in viral hepatitis, mostly focusing on hepatitis C or B [17,18,19,20,21,22,24,25,26] and less on hepatitis D [20,23]. 

As we stated earlier, Romania has a high prevalence of HDV infections [7,13]. However, there are only a few studies from this area that characterize this type of patient, and none that test whether these non-invasive scores could be used as an accessible tool to monitor and assess the progression of liver disease. This study offers additional information on what are the predictive values for severe fibrosis and cirrhosis and for the presence of HCC in HBV/HDV superinfection compared to HBV mono-infection from a “red spot” area in Europe. Given the recent intense emigration phenomenon, knowing the clinical characteristics of the population in the country of origin is essential. Furthermore, offering a comparative analysis and illustrating the progression of a liver disease resulting from a globally impactful infection, especially in a recently developed country that still has areas characterized by a poor socio-economic status, is equally relevant.

### 4.1. Non-Cirrhosis vs. Cirrhosis

HDV infection led to cirrhosis in app. 70% of cases within 5–10 years, a rate 3–4 times higher than that seen for the mono-HBV infection cases [57,58]. In our study, the trend was similar: 64.5% of CHD patients already had cirrhosis, and 7.2% developed it after a median follow-up of 4.7 years, while 40.8% of the patients in the CHB group had cirrhosis at the baseline and 7.6% developed later after a median follow-up of 4.1 years. As expected, the risk of being diagnosed with cirrhosis in the CHD group was 2.33 higher than in the CHB group in our population study (*p* = 0.012). However, our reported prevalence score for cirrhosis at baseline for HBV-infected patients was higher than that reported by J.V. Barbosa et al., of 12.7% [56].

The main characteristics of patients with cirrhosis are listed in Supplementary Table S1. Cirrhotic patients with CHD in our study tended to be younger than those in the CHB cohort and had more severe liver damage reflected in higher levels of AFP, AST, ALT, and lower PLT blood count. Since the risk of developing cirrhosis is higher in the CHD cohort, these findings are not surprising. The progression to cirrhosis was reported by other studies to develop at a younger age in HDV-infected patients, even 15 years earlier than in those with HBV mono-infection [57,58,59]. AST, ALT, and PLT levels were found to be predictors for cirrhosis in both cohorts; this fact is in line with other studies’ reports [24,60]. It is worth mentioning that AFP level was found to be an independent predictor for cirrhosis only for CHB patients, a parameter previously described as an HCC predictor [61,62]. However, this association between AFP levels and cirrhosis was mentioned in another study in which it was proposed as a biomarker for the liver fibrosis stage [63]. The poor prognosis of the outcome can also be seen in the lower cumulative time survival in the CHD cohort when compared with CHB.

*Non-Invasive Score Prediction for Cirrhosis*

FIB-4 and APRI scores were first designed for HCV cohorts, and their application to HBV- or HDV-infected cohorts has been a controversial topic over time. A lot of studies report a better performance of the FIB-4 index than the APRI score, all based on HBV-infected patients [19,64]. When tested on the HDV-infected cohort, both scores failed to accurately predict cirrhosis in several studies; it is worth mentioning that FIB-4 remained the superior score [20,23]. In our study, FIB-4 was associated with the presence of cirrhosis only in the CHB group and the same for APRI score in the CHD group. Both scores failed to diagnose the presence of cirrhosis at the baseline (AUROC < 0.800). AST/ALT ratio or AAR was mentioned in several studies [24,63] to be associated with cirrhosis, especially in HBV-infected patients. Our findings correspond with previous results, with AAR being associated with the presence of cirrhosis only in the CHB cohort. However, AAR failed to predict or diagnose cirrhosis.

The presence of cirrhosis at the baseline is an independent factor associated with a worse outcome during the follow-up period [36]. Thus, to appreciate the risk of developing cirrhosis during the follow-up, we included only the cirrhosis-free patients at their first admission. Surprisingly, only FIB-4 and APRI scores showed a good prediction for the risk of developing cirrhosis and only in the CHD cohort. Additionally, when adjusted for possible confounders, the AAR score proved to be a very good predictor for developing cirrhosis in the CHD group. In the CHB cohort, none of the tested scores were able to predict cirrhosis development.

### 4.2. Non-HCC vs. HCC

In the vast majority of cases, HCC occurs secondary to cirrhosis, which was described in some studies to be the main cause of HCC and not the viral activity at the beginning of the follow-up [65]. The most recent studies showed increasing rates of developing HCC, 16% in a follow-up period of 3 years, a value higher than that reported in previous studies, which described a progression of the HCC disease between 3 and 15% [36,66,67]. In our study, we found that 23.2% of patients developed HCC in the CHD cohort during a mean follow-up of 4.5 years, a higher rate than that reported in the previously mentioned studies. In the CHB cohort, the development of HCC was significantly lower at 8.5%. As expected, when comparing the cohorts, CHD patients had a 3.03 higher risk of developing HCC than those infected only with HBV, proving the significant impact of the HDV coinfection. The cumulative probabilities of progression to HCC are different in the two cohorts, with a lower survival time for the CHD cohort, supporting once again the poor prognosis for HDV coinfection.

Despite so many studies reporting the HCC development only after the onset of cirrhosis [65,68], in our study, there were few cases of HCC that should not be overlooked without a prior diagnosis of cirrhosis. According to a study about HCC globally [69], usually, these cases are reported in endemic areas such as Eastern Asia and Africa [70] and rarely in other regions [70]. Romania always reported high prevalence rates for HBV and HDV infection, and is thus considered an endemic country for this type of infection [53,71]. The lack of cirrhosis diagnosis may also be explained by additional factors such as poor socio-economic status that can cause late presentation, alcohol consumption that may not be reported in the medical record, and other environmental factors that can contribute to the HCC development. Since our study confirms once again that the presence of HDV is associated with a higher risk of developing HCC when compared to HBV mono-infection, it is important to mention the significant differences between these two cohorts that may influence the liver disease outcome.

The main characteristics of patients with HCC divided into the CHD/CHB groups are listed in Supplementary Table S4. There is a noticeable age difference between the two types of infected patients that developed HCC-CHD patients are younger (median age 55 vs. 59), and this may be due to the accelerating effect of HDV association. These findings are in line with other studies that reported a stronger association between HDV infection and HCC development at a younger age when compared to HBV mono-infection [72,73]. Because of all viral hepatitis, HDV infection has been linked to a more rapid progression of liver disease, these findings were expected to also be obtained in our study and align with other reports [10,11,59]. Commonly, the histological findings, along with the clinical input, indicate a more rapid progression and deterioration of liver lesions associated with a more active and severe liver disease among HDV-infected patients, suggesting that HDV superinfection is an important factor that significantly modifies the course of the HBV infection [59]. Consequently, it is expected to find younger patients who develop cirrhosis in the CHD cohort compared to CHB. In our study, we also observed a significant difference in PLT levels between the two investigated cohorts—the HDV cohort with HCC had lower PLT levels than the HBV mono-infected patients with HCC. Previous studies reported similar results, in which the HDV-HCC cohort was associated with lower PLT values [74]. Since age, male gender, and PLT levels were found to be important prognostic factors in assessing the 5-year HCC risk, they were introduced together with albumin–bilirubin in the formula that generates the aMAP score, which satisfactorily predicts the risk of HCC [25]. The pathogenic substrate for low PLT levels in these viral hepatitis diseases is represented by the platelet consumption due to the coagulation induced by the liver necroinflammation, the hypersplenism associated with PLT phagocytosis, sequestration, and their subsequent reduced circulatory levels, and the decrease in the platelets’ production caused by the specific cytokine environment [75]. These processes are known to be much more severe in the case of HDV/HBV superinfection than in HBV mono-infection [10,11]. In binary logistic regressions, there is an important association of AFP with HCC in both groups, proving to be a strong predictor for HCC, as reported so far by other studies [76]. AFP test is known to be a reliable screening and surveillance test for HCC, even in situations with normal transaminase levels, improving the sensitivity of ultrasonography when used together [77]. This was proven once again in our study, where the AFP level qualified as an independent predictor for HCC development in both cohorts. However, when adjusting for possible confounders (age, gender, and the presence of comorbidities), the AFP level remained a good predictor for HCC only in the CHB group. Also, another independent predictor for HCC was a low level of albumin, found only in the CHD cohort. Albumin was reported to have a potential role in inhibiting the HCC growth; thus, lower albumin was associated with larger tumor diameters, more tumor multifocality, and higher AFP levels [78].

*Non-invasive score prediction for HCC*

It is known that HDV increases the risk of HCC development with the need for liver transplantation and causing liver-related death [56]. Hence, there is a need to develop a non-invasive score that accurately predicts HCC, eliminates complications, and saves waiting time for biopsy results. aMAP score was recently developed to predict the HCC occurrence [25] and has already been validated on a hepatitis C cohort with sustained virologic response [79] and a hepatitis B cohort with acute-on-chronic liver failure [80]. However, in our study, aMAP score proved to be a poor predictor for HCC in both cohorts (Figure 5 and Figure 6).

This study strengthens the fact that HDV infection is an important cause of morbidity for HBV infected patients. According to EASL guidelines updates, NITs should be able to appreciate the prognostic beyond the fibrosis stage and to efficiently monitor the progression of liver disease with or without treatment. The FIB-4 score for the CHB cohort and the APRI score for the CHD cohort proved to have good performances in predicting the development of cirrhosis during follow-up. Regarding the HCC diagnosis or development prediction, aMAP score failed to ascertain this outcome. Importantly, including the presence of comorbidities as confounders in our analysis largely increased the AAR’s predictive value for the risk of developing cirrhosis. Importantly, virological status did not significantly contribute to the risk assessment of cirrhosis or HCC; thus, the therapeutic decision was not affected by the baseline HBsAg levels and HBeAg status. However, the antiviral therapy showed only a marginal effect on improving the predictive values of the investigated non-invasive scores at baseline or the rate of evolution toward cirrhosis or HCC. These results are important since both comorbidities and antiviral treatment may influence the clinical outcome of patients. For instance, the Hepatitis Delta International Intervention Trials (HIDIT-I and HIDIT-II) performed in Germany, Greece, Turkey, and Romania reported key findings about the viral clearance in HDV patients treated with pegylated interferon-alpa-2a in monotherapy or in combination with either adefovir or tenofovir for 48 or 96 weeks: (i) neither nucleotide analogs against hepatitis B had an impact on the HDV-RNA negativity; (ii) extended treatment period to 96 weeks had no additional effect on preventing post-treatment relapse; however, (iii) the majority of patients receiving a 96 weeks treatment had either stable or improved fibrosis scores [10,81]. Overall, the clinical trials and case series confirmed an inhibitory effect of pegylated interferon-alfa on HDV replication ranging between 17% and 47% (studies reviewed by Lisa Sandmann and Heiner Wedemeyer [82]), with a 25% value in a Romanian study investigating its effect when administered as monotherapy [83]. Additionally, the presence of compensated liver cirrhosis is not associated with a lower response to interferon in HBV mono-infection, while for HBV/HVD superinfection, it might even improve the treatment response, as reported by some studies [10]. Importantly, the viral genotype may also influence the clinical outcome and treatment response, and it is widely known that HBV genotypes A and B are associated with a better response than HBV genotypes C and D [29,30]. This is highly relevant for Romania since the few available studies have reported a high frequency of HBV genotype D, over 65% [14,84]. For HDV, genotype 1, which is the most frequent in Europe [85] and the only one reported in Romania [50], is known to be associated with a higher risk of developing cirrhosis and a lower response to interferon therapy than other HDV genotypes [86].

A strong point of this study is that considering the EASL recommendations for monitoring chronic HBV infection in populations at risk for liver fibrosis by evaluating the non-invasive fibrosis scores together with transient elastography [27], our results bring additional connected information about the role of these scores in monitoring the patients with HBV/HDV co-infection, and moreover a possibility of risk stratification and linkage to care for these patients. Antiretroviral therapy in HIV-HBV patients is generally followed by a better evolution of liver fibrosis as detected by liver biopsies; however, a small part of the CHB patients still progress toward liver fibrosis and cirrhosis, often without persistent elevation of liver enzymes [87]. The management of these patients is extremely hard; specific interventions must be associated with frequent testing and the use of simple, non-invasive scores. Since few studies are available for describing the HBV and HDV infection status in Romania, one of the countries with the highest prevalence of HBV infection in Europe, this 8-year retrospective study offers a comprehensive image of the HBV infection situation in our country before the COVID-19 pandemic. The presented results are highly relevant for the public health system and may be used as a reference for further studies.

A weak point of this study is that we did not take into account the viral load for these patients since the viral load quantification was performed via an outsourced molecular laboratory. While the information related to the patient’s age at diagnosis was available, the exact age when the infection was contracted was unknown for some of the patients. Since this is a retrospective study, the antiviral treatment periods showed a high variation and were not included in our analysis. Thus, not considering the exact duration of the antiviral treatment is another limitation of our study. Since we included in the study only the patients admitted into the hospitals, our estimated prevalence of HDV infection may not reflect the actual prevalence in the area. It is well known that the evolution of hepatitis D also depends on the HDV genotype. Because all the patients have the same origin, we supposed that all the patients have the same genotype reported in this area (Eastern Europe): genotype 1 [85]. However, there are no accurate data regarding the exact genotype that circulates in Romania. The level of education may be the leading cause for these high rates of worse outcomes for these patients. There was a high number of mono-infected/co-infected HBV/HDV patients excluded from the study because they had only one medical record during the investigated 8 years of follow-up. This high number may be explained by the lack of awareness of the importance of periodic medical examinations. This is why this study raises some concerns and could serve as an initial step in recognizing the importance of screening for viral hepatitis. It may prompt the initiation of national programs aimed to include these patients, ensuring comprehensive monitoring and facilitating the proper access to treatment.

## 5. Conclusions

In conclusion, both in the cirrhosis group and among the patients with HCC, CHD patients had a more rapid evolution toward end-stage liver diseases, displaying a 2.33 times higher risk of cirrhosis and a 3.03 higher risk of developing HCC than HBV mono-infected patients. The tested fibrosis scores (FIB-4, APRI score, and AAR) were significantly increased when cirrhosis was present at baseline; still, unexpectedly, only the FIB-4 and APRI scores showed a good prediction for the evolution toward cirrhosis via ROC analysis, but only in the CHD group. aMAP score—the risk predictor for HCC—showed significantly higher values in patients with HCC in both groups (mono- and super-infected) but failed to predict the risk of developing HCC during the follow-up. Non-invasive scores should always be considered for monitoring patients with chronic hepatitis B and hepatitis D, but only when associated with other diagnosis methods. For instance, combining indexes based on serum markers with imaging-based techniques (transient elastography or vibration-controlled transient elastography) may significantly increase their diagnosis accuracy (EASL) [33].

## Figures and Tables

**Table 1:** Calculation formulas for non-invasive scores.

Non-Invasive Test	Formula	Cut-Off Values
FIB-4 index	=[age (years) × AST (U/L)]/[PLT count (10⁹/L) × sqrt’ (ALT(U/L)]	<1.45—Negative predictive value (NPV) 90% advanced fibrosis >3.25—Specificity 97% and positive predictive value (PPV) 65% for advanced fibrosis [32,33]
APRI score	=[(AST (U/L)/AST ULN (U/L))/PLT count (10⁹/L)] × 100	>1.0—Sensitivity 76% and specificity 72% for cirrhosis [33,34]
AAR	=AST(U/L)/ALT(U/L)	>1.0—in CHB Specificity 97.5% and PPV 66.7% for severe fibrosis or cirrhosis [24,35]
aMAP score	=({0.06 × age + 0.89 × gender (Male: 1, Female: 0) + 0.48 × [(log₁₀BLBt × 0.66) + (Alb × −0.085)] − 0.01 × PLT count} + 7.41/14.77 × 100	<50—Sensitivity 85.7–100% for low risk of HCC development [25]

ULN: upper limit of normal; BLBt: total bilirubin; Alb: albumin; sqrt’: square root; ULN for BLBt ≥ 1.2; BLBt (μmol/L); Alb (g/L); PLT count (10^3^/mm^3^).

**Table 2:** Demographical and paraclinical data at baseline—differences between patients with CHB vs. CHD.

Parameter	CHB		CHD		CHB vs. CHD	*p*-Value
N (%)	436 (72.4%)		166 (27.6%)			
Female/Male	153/283 (35.1%/64.9%)		77/89 (46.4%/53.6%)		Male	0.011 *
Rural/Urban	173/263 (39.7%/60.3%)		58/108 (34.9%/65.1%)		Urban	0.285 *
	**Median**	**IQR**	**Median**	**IQR**		
Age	58.0	52.0–63.0	56.0	49.0–59.0	↓	**<0.001 (Z = −4.04) ⸷**
Cholesterol (mg/dL)	163.0	126.0–203.8	149.0	129.0–180.3	↓	**0.016 (Z = −2.41) ⸷**
AFP (IU/mL)	2.0	2.0–5.8	5.0	2.0–15.3	↑	**<0.001 (Z = −5.44) ⸷**
AST (U/L)	41.5	22.0–75.8	67.0	44.0–105.3	↑	**<0.001 (Z = −6.85) ⸷**
ALT (U/L)	35.0	21.0–60.0	57.5	41.8–106.5	↑	**<0.001 (Z = −7.77) ⸷**
GGT (U/L)	45.0	26.0–92.8	56.0	29.0–121.0	↑	**0.048 (Z = −1.98) ⸷**
BLBt (mg/dL)	1.0	1.0–2.0	1.0	1.0–2.0	↑	**0.003 (Z = −2.92) ⸷**
Albumin (g/dL)	4.0	4.0–4.0	4.0	3.0–4.0	↓	**0.005 (Z = −2.81) ⸷**
PT (INR)	1.0	1.0–1.0	1.0	1.0–1.0		0.557 ⸷
PLT (×10^9^/L)	182.5	110.3–239.8	105.5	71.5–151.5	↓	**<0.001 (Z = −7.95) ⸷**

* Chi-square test; ⸷ Mann–Whitney U test; ↑ = Increasing; ↓ = Decreasing.

**Table 3:** Comorbidities at baseline—differences between patients with CHB vs. CHD.

Comorbidity		CHB	CHD	CHB vs. CHD	*p*-Value
Diabetes		76 (17.4%)	17 (10.2%)	↓	0.032 *
	Type 1 diabetes mellitus	10 (2.3%)	3 (1.8%)		0.498 *
	Type 2 diabetes mellitus	66 (15.1%)	14 (8.4%)	↓	0.030 *
NAFLD		60 (13.8%)	20 (12.0%)		0.342 *
Obesity (Body mass index ≥ 30.0 kg/m^2^)		39 (8.9%)	12 (7.2%)		0.310 *
Hypertension		138 (31.7%)	33 (19.9%)	↓	0.005 *
	Essential hypertension	134 (30.7%)	32 (19.3%)	↓	0.006 *
Ischemic cardiac disease		44 (10.1%)	10 (6.0%)		0.077 *
Ischemic stroke		3 (0.7%)	1 (0.6%)		0.694 *
Smoking status		3 (0.7%)	1 (0.6%)		0.694 *

* Chi-square test; ↓ = Decreasing; NAFLD = non-alcoholic fatty liver disease.

**Table 4:** Baseline characteristics for patients with and without end-stage liver diseases: cirrhosis and/or HCC.

	Parameter	No Cirrhosis or HCC	Cirrhosis	HCC	Trend	*p*-Value
**CHB**	N (%)	174 (39.9%)	178 (40.8%)	84 (19.3%)		
	Female/Male	77/97 (44.3%/55.7%)	60/118 (33.7%/66.3%)	16/68 (19.0%/81.0%)	Male	<0.001 *
	Rural/Urban	56/118 (32.2%/67.8%)	78/100 (43.8%/56.2%)	39/45 (46.4%/55.6%)	Urban	0.031 *
		**Median (IQR)**	**Median (IQR)**	**Median (IQR)**		
	Age	58.5 (51.0–63.0)	56.0 (52.0–61.0)	60 (56.0–65.8)	↑↑	**0.002, x^2^(2) = 12.3 ‡**
	Cholesterol (mg/dL)	192.5 (156.8–222.3)	135 (104.8–174.0)	156.5 (123.8–192.0)	↓↑	**<0.001, x^2^(2) = 69.2 ‡**
	AFP (IU/mL)	2.0 (1.0–3.0)	3.0 (2.0–6.0)	8.0 (2.0–184.8)	↑↑	**<0.001, x^2^(2) = 72.5 ‡**
	AST (U/L)	24.0 (19.0–40.0)	47.5 (31.5–87.0)	75.0 (43.3–124.0)	↑↑	**<0.001, x^2^(2) = 99.9 ‡**
	ALT (U/L)	29.0 (18.0–47.0)	35.0 (22.0–59.5)	49.0 (32.8–85.8)	↑↑	**<0.001, x^2^(2) = 32.2 ‡**
	GGT (U/L)	31.0 (18.0–57.3)	50.0 (30.0–93.5)	91.0 (41.3–206.3)	↑↑	**<0.001, x^2^(2) = 61.6 ‡**
	BLBt (mg/dL)	1.0 (0.0–1.0)	1.0 (1.0–3.0)	1.0 (1.0–2.0)	↑↓	**<0.001, x^2^(2) = 66.7 ‡**
	Albumin (g/dL)	4.0 (4.0–4.0)	4.0 (3.0–4.0)	4.0 (3.0–4.0)	↓↑	**<0.001, x^2^(2) = 44.7 ‡**
	PT (INR)	1.0 (1.0–1.0)	1.0 (1.0–1.0)	1.0 (1.0–1.0)	↑↓	**<0.001, x^2^(2) = 27.6 ‡**
	PLT (×10⁹/L)	223.5 (184.5–266.3)	113.0 (74.8–184.5)	172.5 (116.3–240.8)	↓↑	**<0.001, x^2^(2) = 116.7 ‡**
**CHD**	N (%)	31 (18.7%)	107 (64.5%)	28 (16.9%)		
	Female/Male	18/13 (58.1%/41.9%)	52/55 (48.6%/51.4%)	7/21 (25%/75%)	Male	0.030 *
	Rural/Urban	10/21 (32.3%/67.7%)	39/68 (36.4%/65.6%)	9/19 (32.1%/67.9%)	Urban	0.861 *
		**Median (IQR)**	**Median (IQR)**	**Median (IQR)**		
	Age	56.0 (47.0–59.0)	55.0 (48.0–58.0)	59.0 (55.3–60.8)	↑↑	**0.007 x^2^(2) = 10.1 ‡**
	Cholesterol (mg/dL)	175.0 (149.0–198.0)	142.0 (125.0–166.0)	163.0 (126.8–192.8)	↓↑	**<0.001, x^2^(2) = 17.5 ‡**
	AFP (IU/mL)	3.0 (2.0–9.0)	4.0 (3.0–10.0)	18.5 (5.0–168.3)	↑↑	**<0.001, x^2^(2) = 16.1 ‡**
	AST (U/L)	41.0 (25.0–81.0)	74.0 (52.0–103.0)	81.0 (49.3–138.8)	↑↑	**0.001, x^2^(2) = 13.3 ‡**
	ALT (U/L)	49.0 (26.0–91.0)	57.0 (43.0–109.0)	66.5 (39.3–150.0)	↑↑	0.156, x^2^(2) = 3.7 ‡
	GGT (U/L)	40.0 (21.0–65.0)	60.0 (30.0–126.0)	70.0 (38.0–232.3)	↑↑	**0.006, x^2^(2) = 10.1 ‡**
	BLBt (mg/dL)	1.0 (1.0–1.0)	1.0 (1.0–2.0)	1.0 (1.0–2.8)	↑↓	**<0.001, x^2^(2) = 19.6 ‡**
	Albumin (g/dL)	4.0 (4.0–4.0)	4.0 (3.0–4.0)	4.0 (3.0–4.0)	↓↑	**0.003, x^2^(2) = 11.4 ‡**
	PT (INR)	1.0 (1.0–1.0)	1.0 (1.0–1.0)	1.0 (1.0–1.8)	↑↑	**0.019, x^2^(2) = 7.9 ‡**
	PLT (×10⁹/L)	190.0 (130.0–258.0)	86.0 (61.0–128.0)	116.5 (75.0–161.3)	↓↑	**<0.001, x^2^(2) = 34.7 ‡**

* Chi-square test; ‡ Kruskal–Wallis Test; ↑↑—In the *Trend* column, the first arrow is for the trend between “No cirrhosis or HCC” and “Cirrhosis” groups, and the second one is for the trend between “Cirrhosis” and “HCC” groups. ↑ = Increasing; ↓ = Decreasing.

**Table 5:** Baseline comorbidities for patients with and without end-stage liver diseases: cirrhosis and/or HCC.

	Comorbidity		No Cirrhosis Nor HCC	Cirrhosis	HCC	Trend	*p*-Value
**CHB**	**N (%)**		**174 (39.9%)**	**178 (40.8%)**	**84 (19.3%)**		
	Diabetes		29/145 16.7%/83.3%	29/149 16.3%/83.7%	18/66 21.4%/78.6%	-↑	0.559 *
		Type 1 diabetes mellitus	2/172 1.1%/98.9%	6/172 3.4%/96.6%	2/82 2.4%/97.6%	↑↓	0.379 *
		Type 2 diabetes mellitus	27/147 15.5%/84.5%	23/155 12.9%/87.1%	16/68 19.0/81.0%	↓↑	0.427 *
	NAFLD		43/131 24.7%/75.3%	12/166 6.7%/93.3%	5/79 6.0%/94.0%	↓-	<0.001 *
	Obesity (Body mass index ≥ 30.0 kg/m^2^)		23/151 13.2%/86.8%	12/166 6.7%/93.3%	4/80 4.8%/95.2%	↓↓	0.034 *
	Hypertension		67/107 38.5%/61.5%	46/132 25.8%/74.2%	25/59 29.8%/70.2%	↓↑	0.035 *
		Essential hypertension	64/110 36.8%/63.2%	45/133 25.3%/74.7%	25/59 29.8%/70.2%		0.064 *
	Ischemic cardiac disease		20/154 11.5%/88.5%	16/162 9.0%/91.0%	8/76 9.5%/90.5%	↓-	0.724 *
	Ischemic stroke		0	2/176 1.1%/98.9%	1/83 1.2%/98.8%	↑-	0.366 *
	Smoking status		2/172 1.1%/98.9%	1/177 0.6%/99.4%	0	↓↓	0.558 *
**CHD**	**N (%)**		**31 (18.7%)**	**107 (64.5%)**	**28 (16.9%)**		
	Diabetes		0	16/91 15.0%/85.0%	1/27 3.6%/96.4%	↑↓	0.024 *
		Type 1 diabetes mellitus	0	3/104 2.8%/97.2%	0	↑↓	0.431 *
		Type 2 diabetes mellitus	0	13/94 12.1%/87.9%	1/27 3.6%/96.4%	↑↓	0.060 *
	NAFLD		8/23 25.8%/74.2%	7/100 6.5%/93.5%	5/23 17.9%/82.1%	↓↑	0.009 *
	Obesity (Body mass index ≥ 30.0 kg/m^2^)		4/27 12.9%/87.1%	8/99 7.5%/92.5%	0	↓↓	0.159 *
	Hypertension		10/21 32.3%/67.7%	17/90 15.9%/84.1%	6/22 21.4%/78.6%	↓↑	0.129 *
		Essential hypertension	10/21 32.3%/67.7%	16/91 15.0%/85.0%	6/22 21.4%/78.6%	↓↑	0.094 *
	Ischemic cardiac disease		4/27 12.9%/87.1%	3/104 2.8%/97.2%	3/25 10.7%/89.3%	↓↑	0.060 *
	Ischemic stroke		1/30 3.2%/96.8%	0	0	↓-	0.112 *
	Smoking status		0	1/106 0.9%/99.1%	0	↑↓	0.758 *

* Chi-square test; ↑↑—In the *Trend* column, the first arrow is for the trend between “No cirrhosis or HCC” and “Cirrhosis” groups, and the second one is for the trend between “Cirrhosis” and “HCC” groups. ↑ = Increasing; ↓ = Decreasing; - = no change.

**Table 6:** Non-invasive score prediction at the 1st recorded admission.

	Score	CHB	CHD	CHB vs. CHD	*p*-Value
**Cirrhosis/advanced fibrosis prediction at the 1st admission**	FIB-4 index > 3.25	175 (40.1%)	107 (64.5%)	↑	<0.001 *
	APRI score > 1	158 (36.2%)	114 (68.7%)	↑	<0.001 *
	AST/ALT ratio < 1	253 (58.03%)	96 (57.83%)	↓	0.519 *

* Chi-square test; ↑ = Increasing; ↓ = Decreasing.

**Table 7:** Non-invasive score prediction at 1st recorded admission.

	Score	CHB	CHD	*p*-Value
**Low risk of HCC at the 1st admission**	aMAP score < 50	79 (18.1%)	18 (10.8%)	0.035 *

* Chi-square test.

**Table 8:** Statistical evaluation of non-invasive scores in association with therapy and additional confounders in assessing the risk of developing cirrhosis.

Cohort	Non-Invasive Score	Additional Parameter	AUC	Std. Error	95% CI	*p*-Value
**CHB**	FIB-4	None	0.721	0.056	0.611–0.830	**<0.001**
		Therapy	0.724	0.056	0.614–0.835	**<0.001**
		Therapy + confounders	0.768	0.048	0.675–0.862	**<0.001**
	APRI	None	0.699	0.055	0.591–0.806	**<0.001**
		Therapy	0.709	0.054	0.603–0.815	**<0.001**
		Therapy + confounders	0.775	0.047	0.682–0.867	**<0.001**
	AAR	None	0.574	0.052	0.473–0.676	0.177
		Therapy	0.597	0.054	0.490–0.703	0.079
		Therapy + confounders	0.675	0.051	0.575–0.775	**0.001**
**CHD**	FIB-4	None	**0.824**	0.082	0.663–0.984	**0.003**
		Therapy	0.770	0.093	0.588–0.952	**0.015**
		Therapy + confounders	**0.838**	0.073	0.694–0.982	**0.002**
	APRI	None	**0.877**	0.064	0.752–1.000	**0.001**
		Therapy	**0.853**	0.070	0.716–0.990	**0.001**
		Therapy + confounders	**0.941**	0.040	0.863–1.000	**<0.001**
	AAR	None	0.392	0.107	0.182–0.603	0.330
		Therapy	0.667	0.104	0.463–0.870	0.132
		Therapy + confounders	**0.828**	0.076	0.680–0.977	**0.003**

CHB confounders for FIB-4 (gender, levels of AFP, diabetes, NAFLD, obesity, smoking status, essential hypertension, history of ischemic cardiac disease); APRI (age, gender, levels of ALT and AFP, diabetes, NAFLD, obesity, smoking status, essential hypertension, history of ischemic cardiac disease); AAR (age, gender, levels of AFP, diabetes, NAFLD, obesity, smoking status, essential hypertension, history of ischemic cardiac disease). CHD confounders for FIB-4 (gender, levels of GGT, diabetes, NAFLD, obesity, smoking status, essential hypertension, history of ischemic cardiac disease); APRI (age, gender, levels of ALT and GGT, diabetes, NAFLD, obesity, smoking status, essential hypertension, history of ischemic cardiac disease); AAR (age, gender, levels of GGT, diabetes, NAFLD, obesity, smoking status, essential hypertension, history of ischemic cardiac disease).

**Table 9:** Statistical evaluation of aMAP in association with therapy and additional confounders in assessing the risk of developing HCC.

Cohort	Non-Invasive Score	Additional Parameter	AUC	Std. Error	95% CI	*p*-Value
**CHB**	aMAP	None	0.678	0.039	0.602–0.755	**0.001**
		Therapy	0.686	0.042	0.603–0.768	**0.001**
		Therapy + confounders	0.773	0.047	0.681–0.864	**<0.001**
**CHD**	aMAP	None	0.656	0.055	0.549–0.763	**0.009**
		Therapy	0.656	0.054	0.549–0.762	**0.009**
		Therapy + confounders	0.745	0.048	0.650–0.839	**<0.001**

aMAP confounders for CHB (levels of AST and AFP, diabetes, NAFLD, obesity, smoking status, essential hypertension, history of ischemic cardiac disease) and CHD (levels of AFP, diabetes, NAFLD, obesity, smoking status, essential hypertension, history of ischemic cardiac disease).

## Data Availability

Data are contained within the article.

## Supplementary Materials

The following supporting information can be downloaded at https://www.mdpi.com/article/10.3390/microorganisms11122895/s1. Figure S1: ROC curves of the non-invasive FIB-4 score adjusted for confounders in assessing the risk of developing cirrhosis during follow-up; Figure S2: ROC curves of the non-invasive APRI score adjusted for confounders in assessing the risk of developing cirrhosis during follow-up; Figure S3: ROC curves of non-invasive scores adjusted for the HBsAg levels and HBeAg status for predicting the risk of developing cirrhosis in CHB; Figure S4: ROC curves of non-invasive scores adjusted for the HBsAg levels and HBeAg status for predicting the risk of developing cirrhosis in CHD; Figure S5: ROC curves of the non-invasive aMAP score adjusted for the HBsAg levels and HBeAg status in assessing the risk of developing HCC during follow-up; Table S1: Comparison between patients with cirrhosis from CHB vs. CHD group (baseline characteristics); Table S2: Independent predictors for cirrhosis development; Table S3: Statistical evaluation of non-invasive scores and possible confounders in association with cirrhosis; Table S4: Comparison between HCC patients with CHB vs. CHD (baseline characteristics); Table S5: Independent predictors for HCC; and Table S6: Statistical evaluation of non-invasive scores and possible confounders in association with HCC.
