# Supplementary material for: Non-Invasive Prediction Scores for Hepatitis B Virus- and Hepatitis D Virus-Infected Patients—A Cohort from the North-Eastern Part of Romania
Source: Microorganisms. 2023 Nov 30;11(12):2895. doi: 10.3390/microorganisms11122895 (PMC10745361; doi:10.3390/microorganisms11122895)
Supplement: Supplementary file 1 [file microorganisms-11-02895-s001.zip › microorganisms-2723203-supplementary.pdf]

Supplementary files

Supplementary Figure S1

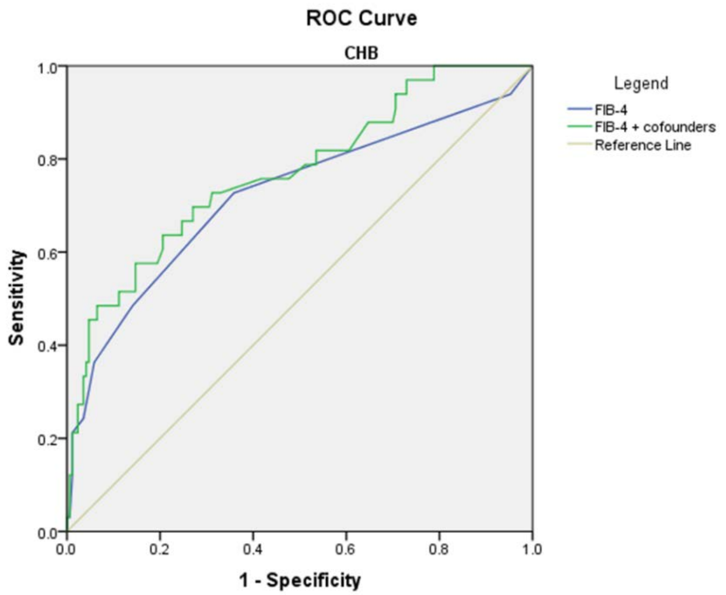

(**a**)

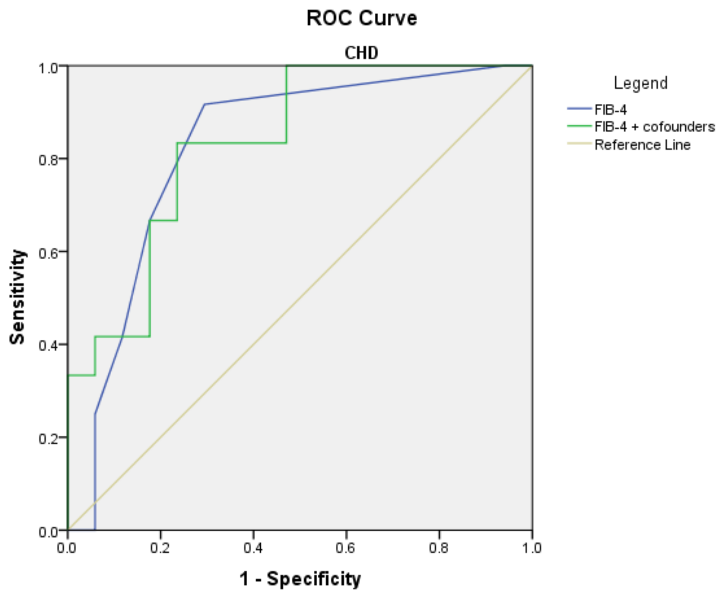

(**b**)

Supplementary Figure S1. ROC curves of the non-invasive FIB-4 score adjusted for confounders in assessing the risk of developing cirrhosis during follow-up: (**a**) CHB; confounders: gender, levels of AFP, diabetes, NAFLD, obesity, smoking status, essential hypertension, history of ischemic cardiac disease; (**b**) CHD; confounders: gender, levels of GGT, diabetes, NAFLD, obesity, smoking status, essential hypertension, history of ischemic cardiac disease.

Supplementary Figure S2

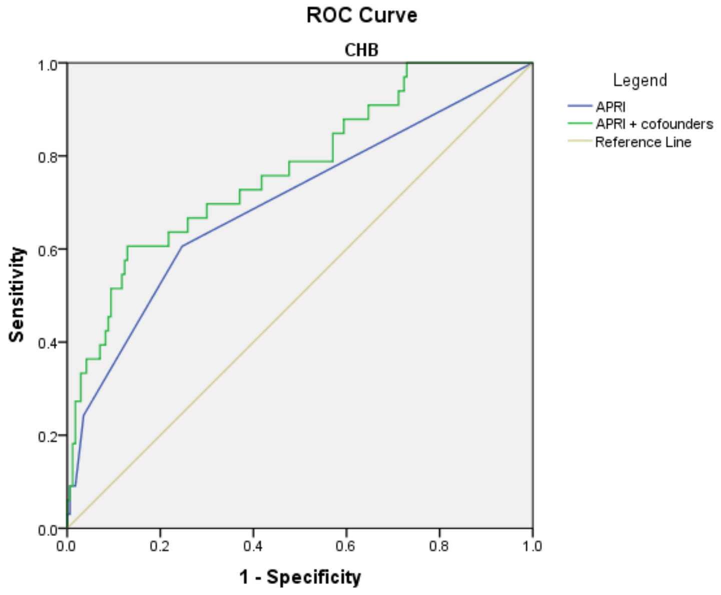

(**a**)

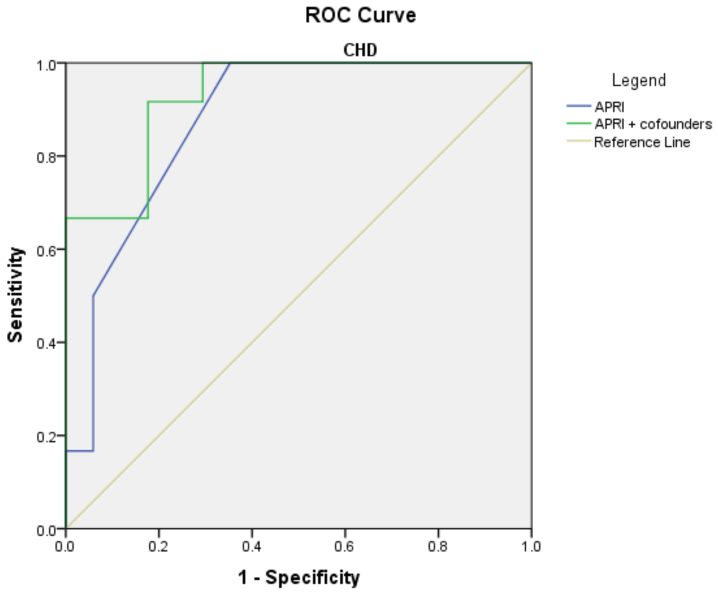

(**b**)

Supplementary Figure S2. ROC curves of the non-invasive APRI score adjusted for confounders in assessing the risk of developing cirrhosis during follow-up: (**a**) CHB; confounders: age, gender, levels of ALT and AFP, diabetes, NAFLD, obesity, smoking status, essential hypertension, history of ischemic cardiac disease; (**b**) CHD; confounders: age, gender, levels of ALT and GGT, diabetes, NAFLD, obesity, smoking status, essential hypertension, history of ischemic cardiac disease.

Supplementary Figure S3

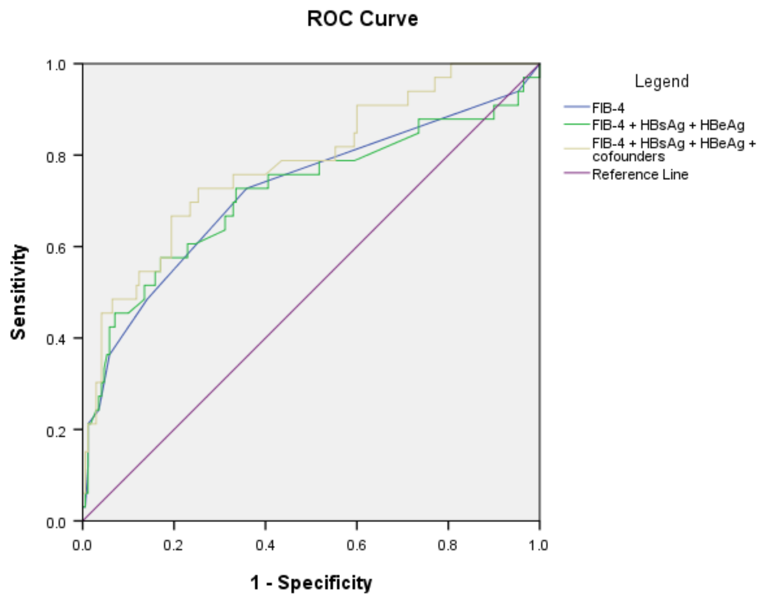

(a)

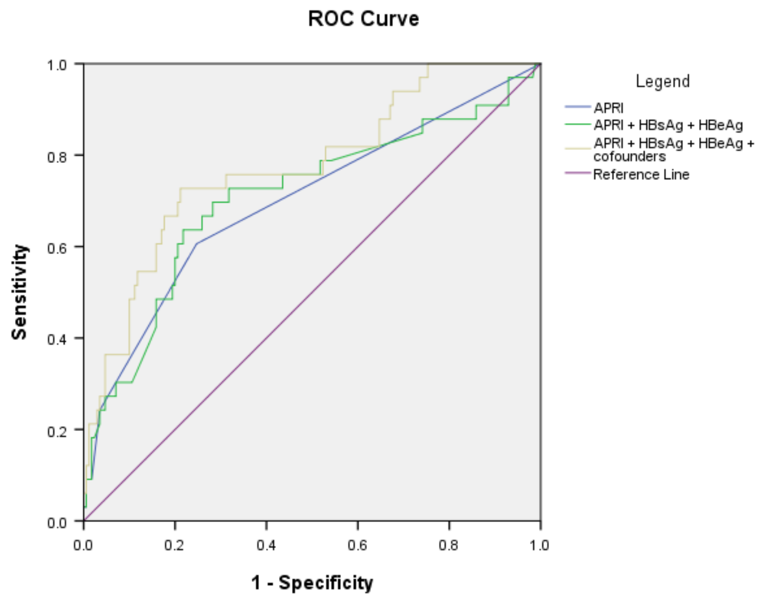

(b)

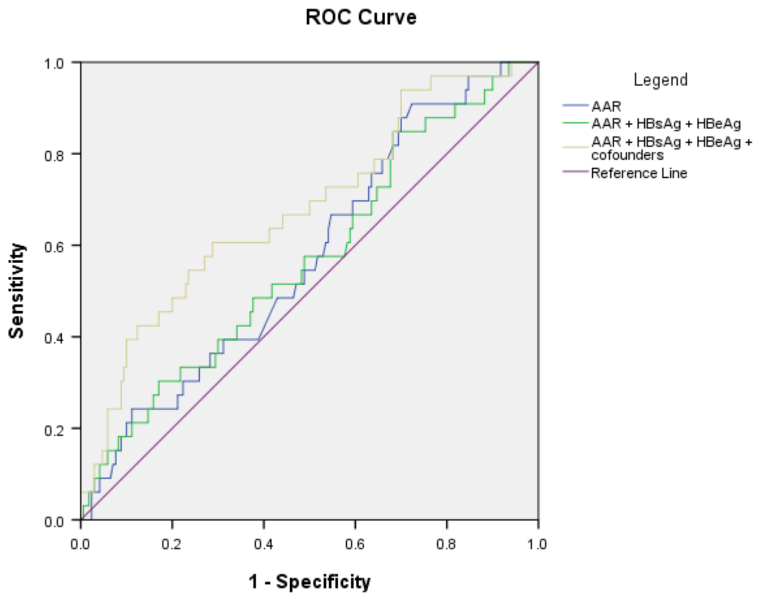

(c)

Supplementary Figure S3. ROC curves of non-invasive scores adjusted for the HBsAg levels and HBeAg status for predicting the risk of developing cirrhosis in CHB: (**a**) FIB-4 (confounders: gender, levels of AFP, diabetes, NAFLD, obesity, smoking status, essential hypertension, history of ischemic cardiac disease); (**b**) APRI (confounders: age, gender, levels of ALT and AFP, diabetes, NAFLD, obesity, smoking status, essential hypertension, history of ischemic cardiac disease); (**c**) AAR (confounders: age, gender, levels of AFP, diabetes, NAFLD, obesity, smoking status, essential hypertension, history of ischemic cardiac disease).

Supplementary Figure S4

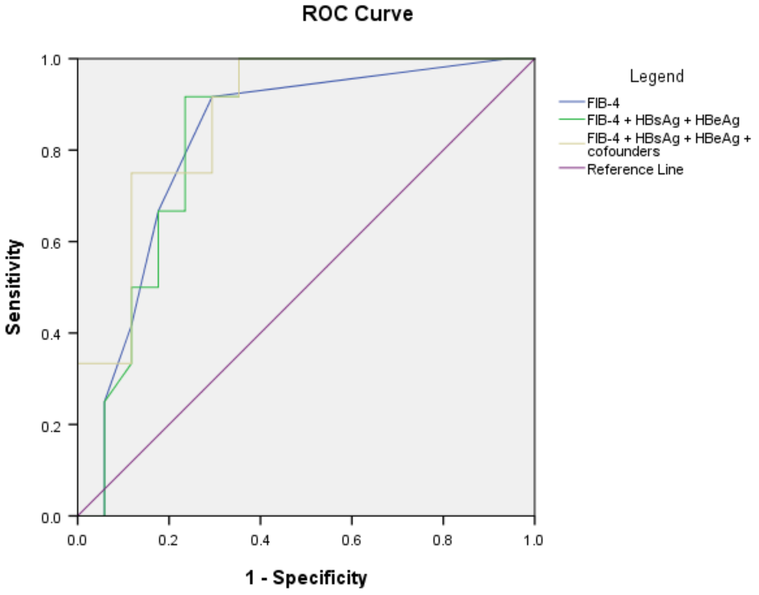

(a)

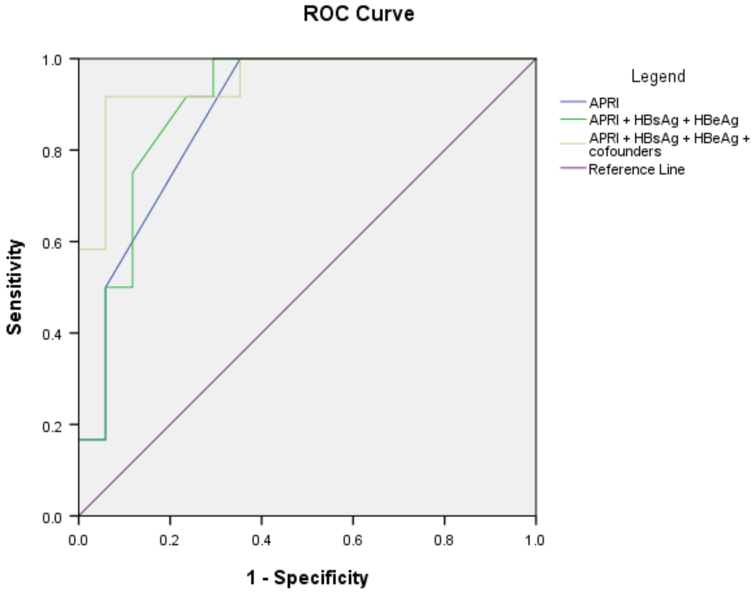

(b)

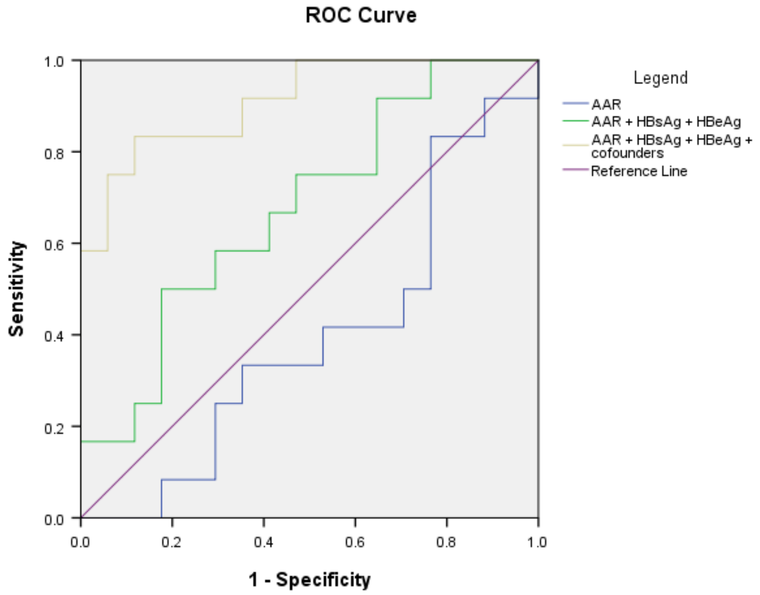

(c)

Supplementary Figure S4. ROC curves of non-invasive scores adjusted for the HBsAg levels and HBeAg status for predicting the risk of developing cirrhosis in CHD: (**a**) FIB-4 (confounders: gender, levels of GGT, diabetes, NAFLD, obesity, smoking status, essential hypertension, history of ischemic cardiac disease); (**b**) APRI (confounders: age, gender, levels of ALT and GGT, diabetes, NAFLD, obesity, smoking status, essential hypertension, history of ischemic cardiac disease); (**c**) AAR (confounders: age, gender, levels of GGT, diabetes, NAFLD, obesity, smoking status, essential hypertension, history of ischemic cardiac disease).

Supplementary Figure S5

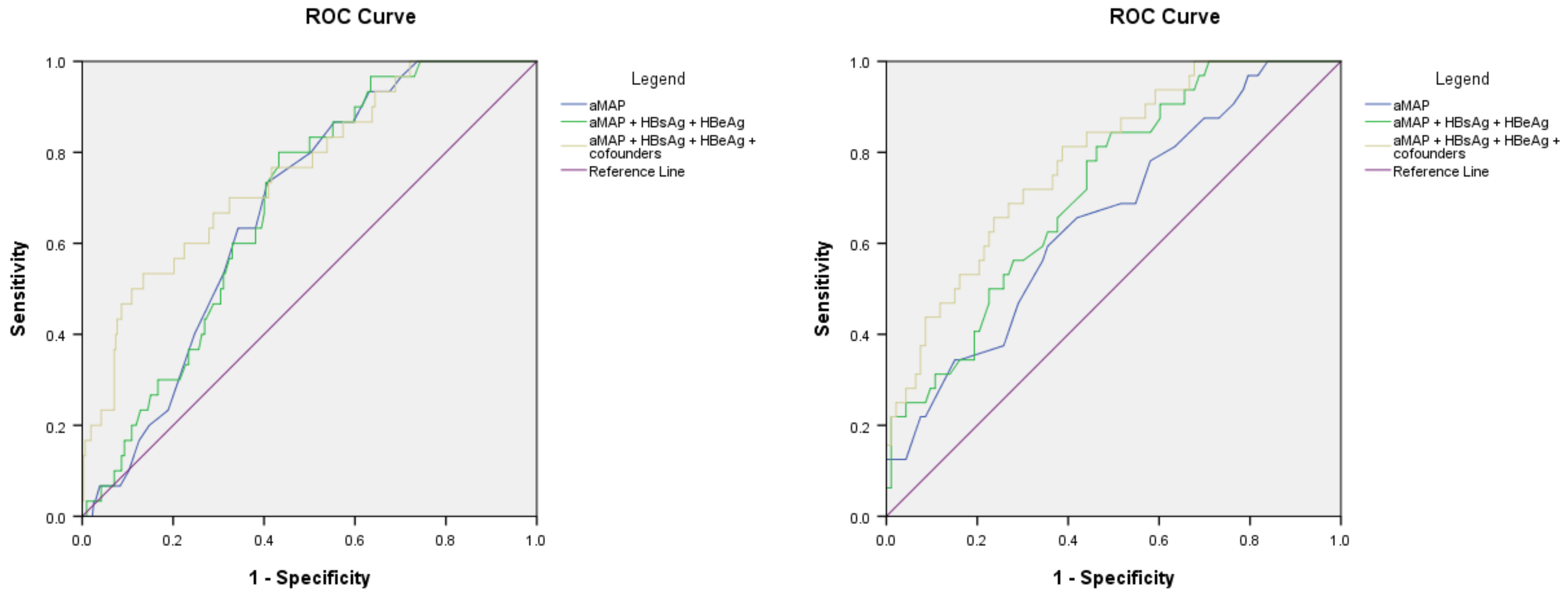

(**a**) (**b**)

Supplementary Figure S5. ROC curves of the non-invasive aMAP score adjusted for the HBsAg levels and HBeAg status in assessing the risk of developing HCC during follow-up: (**a**) CHB; confounders: levels of AST and AFP, diabetes, NAFLD, obesity, smoking status, essential hypertension, history of ischemic cardiac disease; (**b**) CHD; confounders: levels of AFP, diabetes, NAFLD, obesity, smoking status, essential hypertension, history of ischemic cardiac disease.

Supplementary Table S1. Comparison between patients with cirrhosis from CHB *vs.* CHD group (baseline characteristics)

	CHB - cirrhosis		CHD - cirrhosis		CHB *vs.* CHD	*p*-value
	**Median**	**IQR**	**Median**	**IQR**		
**Age**	57.0	52.0 - 63.0	56.0	50.0 - 59.8	↓	**.003 (Z = -3.02)**ϯ
**Cholesterol (mg/dL)**	139.5	107.8 - 177.0	142.5	125.3 - 172.8	↑	.585ϯ
**AFP (UI/mL)**	**3.0**	**2.0 - 9.0**	**5.0**	**3.0 - 15.8**	↑	**.012 (Z = -2.49)**ϯ
**AST (U/L)**	**55.0**	**33.8 - 99.0**	**74.0**	**51.0 - 116.8**	↑	**.001 (Z = -3.24)**ϯ
**ALT (U/L)**	**41.0**	**23.0 - 67.3**	**58.5**	**42.3 - 111.3**	↑	**< .001 (Z = -5.69)**ϯ
**GGT (U/L)**	55.0	31.8 - 122.3	60.5	33.5 - 130.5		.987ϯ
**BLBt (mg/dL)**	1.0	1.0 - 3.0	1.0	1.0 - 2.8		.875ϯ
**Albumin (g/dL)**	4.0	3.0 - 4.0	4.0	3.0 - 4.0		.503ϯ
**PT (INR)**	1.0	1.0 - 1.0	1.0	1.0 - 1.0		.396ϯ
**PLT ($x10^9$/L)**	**118.0**	**78.0 - 188.3**	**90.5**	**64.5 - 139.0**	↓	**< .001 (Z = -4.06)**ϯ

ϯMann-Whitney U test
↑ = Increasing
↓ = Decreasing

Supplementary Table S2. Independent predictors for cirrhosis development

	Independent predictor for cirrhosis	Exp (B)	95% CI for Exp (B)	*p*-value	No cirrhosis *vs.* cirrhosis
**CHB**	**AFP (UI/mL)**	1.002<br>AHR: 1.002	1.001 – 1.003<br>1.001 – 1.003	.001¥<br>.004⁂	↑
	**AST (U/L)**	1.008<br>AHR: 1.010	1.005 – 1.011<br>1.006 – 1.014	< .001¥<br>< .001⁂	↑
	**ALT (U/L)**	1.003<br>AHR: 1.005	1.001 – 1.005<br>1.002 – 1.007	.005¥<br>.001⁂	↑
	**PLT ($x10^9$/L)**	.992<br>AHR: .993	.987 - .998<br>.987 - .998	.007¥<br>.012⁂	↓
**CHD**	**AST (U/L)**	1.020<br>AHR: 1.022	1.008 – 1.032<br>1.009 – 1.036	.001¥<br>001⁂	↑
	**ALT (U/L)**	1.011<br>AHR: 1.012	1.004 – 1.017<br>1.004 – 1.019	.001¥<br>.004⁂	↑
	**GGT (U/L)**	1.004<br>AHR: 1.007	1.001 - 1.007<br>1.001 - 1.012	.010¥<br>.013⁂	↑
	**PLT ($x10^9$/L)**	.988<br>AHR: .984	.978 - .997<br>.972 - .997	.012¥<br>.013⁂	↓

¥Univariable Cox proportional-hazards regression
⁂Multivariable Cox proportional-hazards regression (adjusted for possible confounders: age, gender, diabetes, NAFLD, obesity, smoking status, essential hypertension, history of ischemic cardiac disease)

Supplementary Table S3. Statistical evaluation of non-invasive scores and possible confounders in association with cirrhosis

	**Non-invasive score**	**AUC**	**Std. error**	**95% CI**	***p* - value**
**CHB**	FIB-4	0.721	0.056	0.611 – 0.830	< **.001**
	FIB-4 + confounders	0.769	0.048	0.675 – 0.862	< **.001**
ROC curve for FIB-4 in the CHB cohort adjusted for possible confounders: gender, levels of AFP, diabetes, NAFLD, obesity, smoking status, essential hypertension, history of ischemic cardiac disease.					
**CHD**	FIB-4	**0.824**	0.082	0.663 – 0.984	**.003**
	FIB-4 + confounders	**0.833**	0.074	0.688 – 0.979	**.003**
ROC curve for FIB-4 in the CHD cohort adjusted for possible confounders: gender, levels of GGT, diabetes, NAFLD, obesity, smoking status, essential hypertension, history of ischemic cardiac disease.					
**CHB**	APRI	0.699	0.055	0.591 – 0.806	< **.001**
	APRI + confounders	0.770	0.047	0.679 – 0.862	< **.001**
ROC curve for APRI in the CHB cohort adjusted for possible confounders: age, gender, levels of ALT and AFP, diabetes, NAFLD, obesity, smoking status, essential hypertension, history of ischemic cardiac disease.					
**CHD**	APRI	**0.877**	0.064	0.752 – 1.000	**.001**
	APRI + confounders	**0.931**	0.044	0.844 – 1.000	< **.001**
ROC curve for APRI in the CHD cohort adjusted for possible confounders: age, gender, levels of ALT and GGT, diabetes, NAFLD, obesity, smoking status, essential hypertension, history of ischemic cardiac disease.					
**CHB**	AAR	0.574	0.052	0.473 – 0.676	.177
	AAR + confounders	0.687	0.054	0.581 – 0.793	**.001**
ROC curve for AAR in the CHB cohort adjusted for possible confounders: age, gender, levels of AFP, diabetes, NAFLD, obesity, smoking status, essential hypertension, history of ischemic cardiac disease.					
**CHD**	AAR	0.392	0.107	0.182 – 0.603	**.330**
	AAR + confounders	**0.863**	0.066	0.732 – 0.993	**.001**
ROC curve for AAR in the CHD cohort adjusted for possible confounders: age, gender, levels of GGT, diabetes, NAFLD, obesity, smoking status, essential hypertension, history of ischemic cardiac disease.					

Supplementary Table S4. Comparison between HCC patients with CHB *vs.* CHD (baseline characteristics)

	CHB		CHD		CHB *vs.* CHD	*p*-value
	Mean ± SD		Mean ± SD			
**Age**	60.6 ± 8.4		58.3 ±5.3		↓	**.022‡**
**Cholesterol (mg/dl)**	163.4 ± 55.2		157.0 ± 51.8			.980‡
	**Median**	**IQR**	**Median**	**IQR**		
**AFP (UI/mL)**	8.0	2.0 - 184.8	18.5	5.0 - 168.3		.497†
**AST (U/L)**	75.0	43.3 - 124.0	81.0	49.3 - 138.8		.519†
**ALT (U/L)**	49.0	32.8 - 85.8	66.5	39.3 - 150.0		.179†
**GGT (U/L)**	91.0	41.3 - 206.3	70.0	38.0 - 232.3		.450†
**BLBt (mg/dL)**	1.0	1.0 - 2.0	1.0	1.0 - 2.8		.752†
**Albumin (g/dL)**	4.0	3.0 - 4.0	3.0	3.0 - 4.0		.759†
**PT (INR)**	1.0	1.0 - 1.0	1.0	1.0 - 1.8		.256†
**PLT ($x10^9$/L)**	**172.5**	**116.3 - 240.8**	**116.5**	**75.0 - 161.3**	↓	**.001 (Z = -3028)**†

‡Independent Samples T-Test
†Mann-Whitney U test
↑ = Increasing
↓ = Decreasing

Supplementary Table S5. Independent predictors for HCC

	Independent predictor for HCC	Exp (B)	95% CI for Exp (B)	*p*-value	No HCC *vs.* HCC
**CHB**	**AFP (UI/mL)**	1.002<br>AHR: 1.002	1.001 – 1.003<br>1.001 – 1.003	< .001¥<br>< .001⁂	↑
	**AST (U/L)**	1.004<br>AHR: -	1.000 – 1.008<br>-	.045¥<br>.218⁂	↑
	**PLT (x10⁹/L)**	.994<br>AHR: .993	.989 - .998<br>.988 - .999	.008¥<br>.018⁂	↓
**CHD**	**Age**	1.057<br>AHR: 1.068	1.017 – 1.097<br>1.023 – 1.114	.005¥<br>.003⁂	↑
	**AFP (UI/mL)**	1.010<br>AHR: -	1.005 – 1.015<br>-	< .001¥<br>.070⁂	↑
	**Albumin (g/dL)**	.659<br>AHR: .640	.499 - .870<br>.475 - .862	.003¥<br>.003⁂	↓

¥Univariate Cox regression
⁂Multivariable Cox proportional-hazards regression (adjusted for possible confounders: age, gender, diabetes, NAFLD, obesity, smoking status, essential hypertension, history of ischemic cardiac disease)
↑ = Increasing
↓ = Decreasing

Supplementary Table S6. Statistical evaluation of non-invasive scores and possible confounders in association with HCC

<table>
<tr><th></th><th>Non-invasive score</th><th>AUC</th><th>Std. error</th><th>95% CI</th><th>p - value</th></tr>
<tr><td rowspan="2">CHB</td><td>aMAP</td><td>0.678</td><td>0.039</td><td>0.602 – 0.755</td><td>.001</td></tr>
<tr><td>aMAP + confounders</td><td>0.762</td><td>0.043</td><td>0.678 – 0.846</td><td>< .001</td></tr>
<tr><td colspan="6">ROC curve for aMAP score in the CHB cohort adjusted for possible confounders: levels of AST and AFP, diabetes, NAFLD, obesity, smoking status, essential hypertension, history of ischemic cardiac disease.</td></tr>
<tr><td rowspan="2">CHD</td><td>aMAP</td><td>0.656</td><td>0.055</td><td>0.549 – 0.763</td><td>.009</td></tr>
<tr><td>aMAP + confounders</td><td>0.720</td><td>0.053</td><td>0.616 – 0.825</td><td>< .001</td></tr>
<tr><td colspan="6">ROC curve for aMAP score in the CHD cohort adjusted for possible confounders: levels of AFP, diabetes, NAFLD, obesity, smoking status, essential hypertension, history of ischemic cardiac disease.</td></tr>
</table>
